# Deep learning-based android malware detection under realistic evaluation constraints: an architecture-centric review and reliability-aware empirical validation

**DOI:** 10.3389/frai.2026.1964400

**Published:** 2026-10-14

**Authors:** Syed Shuja Hussain, Mohd Faizal Ab Razak, Idris Ismail, Ahmad Firdaus, Abdul Azeem, Jamil Zara, Shamim Akhter

**Affiliations:** 1 Faculty of Computing, Universiti Malaysia Pahang Al-Sultan Abdullah, Pekan, Pahang, Malaysia; 2 PER5ONA Lab, Faculty of Engineering, Universiti Teknologi PETRONAS, Bandar Seri Iskandar, Malaysia; 3 Huanjiang Laboratory, Shaoxing, Zhejiang, China; 4 Faculty of Computing, Management, and Sciences, Universiti Teknologi PETRONAS, Bandar Seri Iskandar, Malaysia; 5 Department of Electrical Engineering, California State University San Marcos, San Marcos, CA, United States

**Keywords:** android malware detection, CICMalDroid 2020, deep learning, explainable AI, realistic evaluation, reliability-aware validation, robustness

## Abstract

**Introduction:**

Deep learning (DL) has reshaped Android malware detection (AMD) by enabling models to learn from heterogeneous Android artifacts, including permissions, DEX and bytecode representations, API and system-call traces, network traffic, image encodings, and program graphs. However, high benchmark accuracy does not necessarily imply reliable, robust, explainable, reproducible, or deployable malware detection.

**Methods:**

This study examines DL-based AMD under realistic evaluation constraints through an architecture-centric systematic review and reliability-aware empirical validation. A PRISMA-guided review of studies published between 2021 and 2026 was conducted to analyze architecture families, artifact representations, dataset transparency, validation realism, robustness and evasion evidence, explainability, reproducibility, and deployment readiness. Controlled empirical validation was also conducted on CICMalDroid 2020 using representative CNN, GRU, Transformer, and Hybrid CNN–LSTM models.

**Results:**

The review shows that DL-based AMD is architecturally diverse, but its supporting evidence remains incomplete. Most studies report datasets, feature sources, model architectures, and conventional metrics, whereas robustness testing, artifact-grounded explainability, reproducibility assets, and deployment evidence remain inconsistent. The empirical validation shows that although all models achieve strong clean cross-validation performance, class-wise analysis, malware false-negative behavior, repeated-run stability, and multi-level test-time feature masking reveal architecture-dependent reliability differences that are not captured by aggregate accuracy.

**Discussion:**

The study contributes an architecture–artifact–validation triad and a claim-aligned reliability audit to align AMD claims with the maturity of supporting evidence. Overall, the findings support a transition from accuracy-centric benchmarking to reliability-centric evaluation, where models are assessed using credible datasets, realistic validation, robustness, interpretability, reproducibility, and operational feasibility.

## 1 Introduction

Android malware detection (AMD) remains a critical problem in mobile security. Android applications operate within a large, open, and continuously evolving software ecosystem. Malicious applications exploit these systems through repackaging, permission abuse, code obfuscation, dynamic payload loading, runtime activation, network communication, and adversarial manipulation. Therefore, AMD should not only be treated as a conventional classification task (Dahiya et al., 2025). Instead, it should be treated as an evidence-centered security problem. In this, the credibility of a detection claim depends on what Android evidence is observed, how it is represented, and which architecture learns from it. It also depends on whether the validation protocol reflects realistic constraints (Multani et al., 2026; Razgallah et al., 2021).

Conventional AMD methods relied on signature-based detection, static/dynamic analysis, and conventional machine-learning (ML) pipelines. These methods proved significant, but each has clear operational limitations (Multani et al., 2026). Signature-based methods seem efficient but show weakness against novel, repackaged, or obfuscated malware. Static methods support fast pre-installation screening but may fail when malware is hidden or structurally transformed. Dynamic analysis can reveal runtime behavior, but its effectiveness depends on whether that behavior is triggered during observation. Hybrid and ML-based approaches combine multiple evidence sources but are affected by feature engineering, bias, and reproducibility limitations (Razgallah et al., 2021). These constraints have motivated the rapid adoption of deep learning (DL), which can learn representations from heterogeneous Android artifacts rather than relying only on manually engineered features (Zhu et al., 2023).

Recent DL-based AMD studies published between 2021–2026 show significant architectural development. Convolutional neural networks (CNNs) are used to learn from Android artifacts, into DEX/bytecode/permissions/Markov images, or multi-channel APK representations (Tasyurek and Arslan, 2023). Sequential methods (LSTM, GRU, and BiGRU) are applied to API-call sequences, system call traces, and flow of HTTP or TCP (Ullah et al., 2024). Transformer and attention-based models have further enhanced contextual learning to model structural dependencies (Ullah et al., 2022a; Jo et al., 2023; Tang et al., 2026). In parallel, hybrid, lightweight, transfer learning, and explainable AI-dependent models have emerged to address the limitations of existing models (i.e., representation capacity, computational efficiency, and interpretability) (Jo et al., 2023).

Although the architecture is continuously growing, it does not imply reliable progress. A CNN, BiGRU, GNN, and transformer trained on DEX images, HTTP/TCP traces, function-call graphs, or traffic tokens, respectively, would not solve the same detection problems. Each architecture is shaped by its own input representation and by the validation conditions under which it is tested. Reported accuracies across studies are difficult to compare because of differences in data, features, validation, protocols, classification, distribution, and assumptions. Therefore, the ranking of studies by reported accuracies risks concealing whether a model has learned generalizable malware evidence or only benchmark-specific regularities (Razgallah et al., 2021; Zhu et al., 2023). This makes artifact-to-architecture mapping critical to understand the actual contribution and limitations of recent DL-based AMD studies.

Dataset validity is central to the credibility of empirical evidence in AMD. Widely used datasets such as Drebin and Genome remain historically important, but they may not fully reflect current Android malware behaviors. Newer datasets (such as Google Play, APKPure, VirusShare, CIC MalDroid 2020, and others) and custom corpora extend this evidence base. Still, there are differences in sample age, source verification of benign samples, label quality, and class balance. Empirical studies have shown that feature selection, analysis type, classification design, and time-aware evaluation can significantly affect the performance interpretation (Dahiya et al., 2025; Multani et al., 2026; Razgallah et al., 2021). Thus, the validation protocol is not a minor experimental detail but rather a factor that determines the credibility of evidence (Shar et al., 2023).

Robustness, explainability, reproducibility, and deployment readiness remain important but unevenly addressed constraints (Qiu et al., 2020). Many DL-based AMD models are evaluated mainly on clean benchmark samples, but real Android malware may be obfuscated, repackaged, padded with benign code, dynamically loaded, or adversarially manipulated (Shar et al., 2023; Qiu et al., 2020). Image-based models may learn visual separability but without preserving meaningful malware semantics (Ullah et al., 2024). Sequential models may fail when malicious behavior is not triggered during observation. Graph models may rely only on fragile call-graph extraction only (Tang et al., 2026). Transformer or attention models may provide attention maps that are not necessarily causal explanations (Ullah et al., 2022a). Similarly, explainable AI (XAI) methods such as Grad-CAM, SHAP, LIME, and attention rollout are useful only if their outputs can be mapped to meaningful artifacts of Android (such as APIs, permissions, DEX methods, or others) (Ullah et al., 2022a; Jo et al., 2023). Practical deployment requires evidence on model sizing, inference latency, memory requirements, and computational and reproducibility costs, which are not currently reported in recent studies.

The existing surveys have generated valuable classifications of AMD, ML pipelines, datasets, and static, dynamic, and hybrid analysis (Dahiya et al., 2025; Multani et al., 2026; Razgallah et al., 2021; Zhu et al., 2023). However, the recent literature emphasizes more targeted synthesis due to an inclination toward graph learning, transformers, XAI systems, lightweight, hybrid, and deployment-aware frameworks (Tasyurek and Arslan, 2023; Ullah et al., 2024; Ullah et al., 2022a; Jo et al., 2023; Shar et al., 2023; Qiu et al., 2020; Bourebaa and Benmohammed, 2025). What remains insufficiently integrated is a review framework that jointly examines architecture evolution, Android artifact representation, empirical credibility, robustness, reproducibility, and deployment readiness. Moreover, most prior reviews do not complement their synthesis with controlled empirical validation showing how model interpretation changed when clean benchmark performance is supplemented with class-wise reliability, repeated-run stability, false-negative behavior, and robustness-oriented testing. To clarify this gap, Table 1 positions the present study against 13 relevant review articles and clarifies the specific gap addressed by this review.

**Table 1:** Review position in comparison to existing AMD surveys.

References	Android specific relevance	PRISMA/SLR protocol	DL architecture focus	Covers (2021–2026)	Dataset validation/evaluation	Robustness/evasion focus	XAI/interpretability focus	Deployment readiness focus	Empirical validation	Remarks/gap addressed
Dahiya et al. (2025)	✓	✓	◐	◐	✓	◐	x	x	x	Recent SLR covering Android malware analysis and detection across static, dynamic, and hybrid methods, datasets, tools, features, models, and metrics; however, it does not provide an architecture-centered DL evidence matrix or controlled reliability-aware empirical validation.
Razgallah et al. (2021)	✓	◐	◐	◐	✓	◐	x	◐	x	Android-specific survey covering static, dynamic, and hybrid detection approaches with attention to datasets, metrics, limitations, and future recommendations; however, it is not an architecture-centered DL synthesis and does not provide reliability-aware empirical validation.
Mehrabi Koushki et al. (2022)	✓	◐	◐	◐	✓	◐	x	◐	x	Valuable pipeline-level review, but not an architecture-centered synthesis of recent DL model families or deployment evidence.
Naway and Li (2018)	✓	◐	✓	x	◐	◐	x	x	x	Review focused on early DL review but predates GNNs, transformers, XAI models, and lightweight deployment frameworks.
Senanayake et al. (2021)	✓	✓	◐	x	✓	◐	x	x	x	Strong ML-oriented SLR, but the DL architectures are not examined as distinct evidence-producing model families.
Kouliaridis and Kambourakis (2021)	✓	◐	◐	x	◐	◐	x	x	x	A broad overview of ML survey type; it does not separate architecture-driven progress from gains caused by feature engineering or dataset choice.
Liu et al. (2022)	✓	✓	✓	◐	◐	◐	x	◐	x	Closest prior DL review, but the evidence window ends before many recent GNN, transformer, XAI, and edge-aware developments.
Manzil and Naik (2024)	✓	◐	◐	◐	✓	◐	x	◐	x	Strong detection classification but not focused on the post-2021 DL architecture shift or credibility.
Jain et al. (2026)	✓	✓	◐	✓	◐	✓	x	◐	x	Focused on hybrid static-dynamic detection modality rather than DL architecture evolution across image, sequence, and XAI models.
Xu et al. (2024)	◐	✓	◐	✓	◐	◐	◐	x	x	Useful for foundational context but not Android- specific. It rarely investigates APK artifacts, Android datasets, or deployment constraints.
Shu et al. (2023)	✓	◐	✓	◐	◐	◐	x	◐	x	Useful CNN-focused AMD survey explaining the CNN detection pipeline, feature extraction, representation, and evaluation; however, it is limited to CNN-based methods and does not synthesize broader DL families such as Transformers, GNNs, hybrid models, and XAI-oriented architectures.
Tanha and Kafaie (2025)	✓	◐	◐	✓	◐	◐	✓	◐	x	Highly relevant XAI-focused review for AMD; however, it focuses mainly on explainability methods and explanation quality rather than architecture-wide reliability, robustness, reproducibility, and deployment-centered empirical validation.
Champie and Elish (2026)	◐	✓	✓	✓	◐	◐	✓	◐	x	Emerging LLM-focused malware detection review with relevance to Transformer/LLM-based detection, explainability, robustness, privacy, and efficiency; however, it is broader than Android AMD and does not provide Android-specific empirical validation or deployment-aware AMD benchmarking.
This review	✓	✓	✓	✓	✓	✓	✓	✓	✓	Provides a PRISMA-guided, architecture-centered, evidence-centered, and deployment-conscious review of DL-based AMD from 2021–2026. It is also complemented by empirical validation using CICMalDroid 2020.

✓: covered, ◐: partially covered, x: not covered.

Accordingly, this study presents a PRISMA-guided architecture-centered review of recent DL-based AMD studies published between 2021 and 2026. This review is complemented by reliability-aware empirical validation using CICMalDroid 2020. The review does not aim to identify which architecture reports the highest accuracy. Instead, it examines whether the evidence supporting DL-based AMD claims is sufficiently mature to justify claims of reliability, robustness, explainability, reproducibility, and deployment feasibility. Empirical validation functions as a controlled reliability audit rather than a state-of-the-art model-ranking exercise. It evaluates representative CNN, GRU, Transformer, and Hybrid (CNN-LSTM) models using clean cross-validation performance, class-wise detection reliability, malware false-negative behavior, repeated-run stability, and 5%, 10%, 20%, and 30% test-time feature masking. Based on this motivation, the study is guided by research questions presented in Table 2.

**Table 2:** Research questions for the study and their rationale.

No.	Research question	Rationale
**1**	How has DL architecture for AMD evolved during 2021–2026?	To examine the shift from conventional DL models toward CNN/image-based, sequential, graph, transformer, attention, hybrid, lightweight, and XAI-based architectures.
**2**	What Android artifacts and feature representations are used by recent DL-based AMD studies?	To map DL architectures to the Android evidence sources they learn from.
**3**	How credible is the empirical evidence supporting recent DL-based AMD studies?	To assess dataset transparency, artifact clarity, architecture reporting, metric adequacy, validation realism, reproducibility, and evidence completeness.
**4**	To what extent do recent studies evaluate robustness, explainability, and reproducibility?	To determine whether DL-based AMD claims are tested beyond clean benchmark accuracy and whether explanations are grounded in Android artifacts.
**5**	What deployment challenges limit the practical use of recent DL-based AMD models?	To evaluate whether studies report model size, inference latency, memory footprint, preprocessing cost, hardware setting, and real-device or edge feasibility.

This study makes four main contributions. First, it provides an architecture-centered synthesis of DL-based AMD research from 2021–2026, distinguishing DL-based AMD-oriented model families, thereby addressing RQ1. Second, it develops an artifact-to-architecture mapping that links each architecture family to the Android evidence it learns from, clarifying why reported performance cannot be interpreted independently of feature representation and validation context, addressing RQ2 and supporting RQ5. Third, it introduces an evidence-credibility assessment framework that evaluates DL-based AMD studies across dataset transparency, artifact clarity, architecture clarity, metric adequacy, validation realism, robustness/evasion evidence, XAI/interpretability, reproducibility, and deployment evidence. This contribution addresses RQ3, RQ4, and RQ5 by shifting the evaluation focus from reported accuracy to the maturity of supporting evidence. Fourth, the study performs controlled reliability-aware empirical validation on CICMalDroid 2020 with representative DL architectures. The empirical part does not propose a new malware detection model but rather shows the change in interpretation of the model when supplementing clean performance with class-wise reliability, malware false-negative behavior, repeated-run stability, and multi-level feature-masking robustness. Together, these contributions provide an architecture-artifact-validation triad and a claim-aligned reliability audit that aligns AMD claims to the evidence that would support them. The rest of the paper is organized as follows. Section 2 describes the methodology, the review protocol according to PRISMA, the criteria for selecting studies, the data extraction strategy, the evidence-credibility framework, and the empirical validation design. Section 3 presents the architecture-driven synthesis and artifact-to-architecture mapping of the recent DL-based AMD studies. Section 4 presents the controlled reliability-aware empirical validation using CICMalDroid 2020. Section 5 discusses reliability-centered evaluation, covering the architecture–artifact–validation triad, claim-aligned reliability audit, and self-audit of the empirical validation. Finally, Section 6 concludes the paper by summarizing the findings, limitations, and directions for future research.

## 2 Methodology

This study adopts a PRISMA-guided systematic literature review (SLR) combined with controlled empirical validation. The review component identifies and synthesizes recent DL-based AMD studies. The empirical component shows why clean benchmark performance should be interpreted together with class-wise reliability, repeated-run stability, and robustness-oriented evidence. This methodological design directly follows the central argument developed in Section 1: “DL-based AMD should be evaluated not only by reported accuracy, but by the maturity of evidence supporting reliability, robustness, explainability, reproducibility, and deployment feasibility”.

### 2.1 Design

This review was designed to examine DL-based AMD studies published between 2021–2026. The rationale for this duration is the transition from conventional DL methods toward GNNs, transformers, XAI, lightweight architectures, adversarial evolution, and deployment awareness in AMD environments. Therefore, this review focused on architecture evolution, Android architecture representation, and the credibility of supporting empirical evidence.

The literature search was conducted across IEEE Xplore, ACM Digital Library, Scopus, Web of Science, Science Direct, Springer, Wiley, and Google Scholar. Google Scholar was used only as a supplementary source for forward and backward snowballing and for identifying recent technical studies not retrieved through main indexed databases. The search strings combined the terms android, DL architecture, and AMD. A representative search expression includes:

“Android” OR “APK” OR “mobile application”) AND (“malware detection” OR “malware classification” OR “malicious app detection”) AND (“DL” OR “CNN” OR “RNN” OR “LSTM” OR “GRU” OR “autoencoder” OR “graph neural network” OR “GNN” OR “transformer” OR “attention” OR “BERT” OR “explainable AI” OR “lightweight model.”

The PRISMA-guided selection process involved five stages, i.e., identification, screening, eligibility, assessment, and final study inclusion. The initial pool included 739 records. After removing 214 duplicate records, 525 records were retained for title and abstract screening. During screening, 421 records were excluded because they were outside the AMD scope, did not include DL or architecture- relevant methods, or were not peer-reviewed. The exclusion also considered articles not available in English or not relevant to the research questions. This stage retained 104 full-text articles for eligibility assessment. At the full-text eligibility stage, 43 articles were excluded. Exclusion reasons included insufficient Android-specific focus, absence of a DL-based detection or representation-learning component, insufficient empirical details, or lack of validation and associated reasons. The final synthesis retained 61 studies. These consisted of 33 primary technical studies, 13 review or survey papers used to position the present review, and 15 other supporting studies used to contextualize datasets, validation, robustness, explainability, reproducibility, and deployment issues. Figure 1 presents the PRISMA-guided selection process, and the inclusion and exclusion criteria are summarized in Table 3.

**Figure 1:**
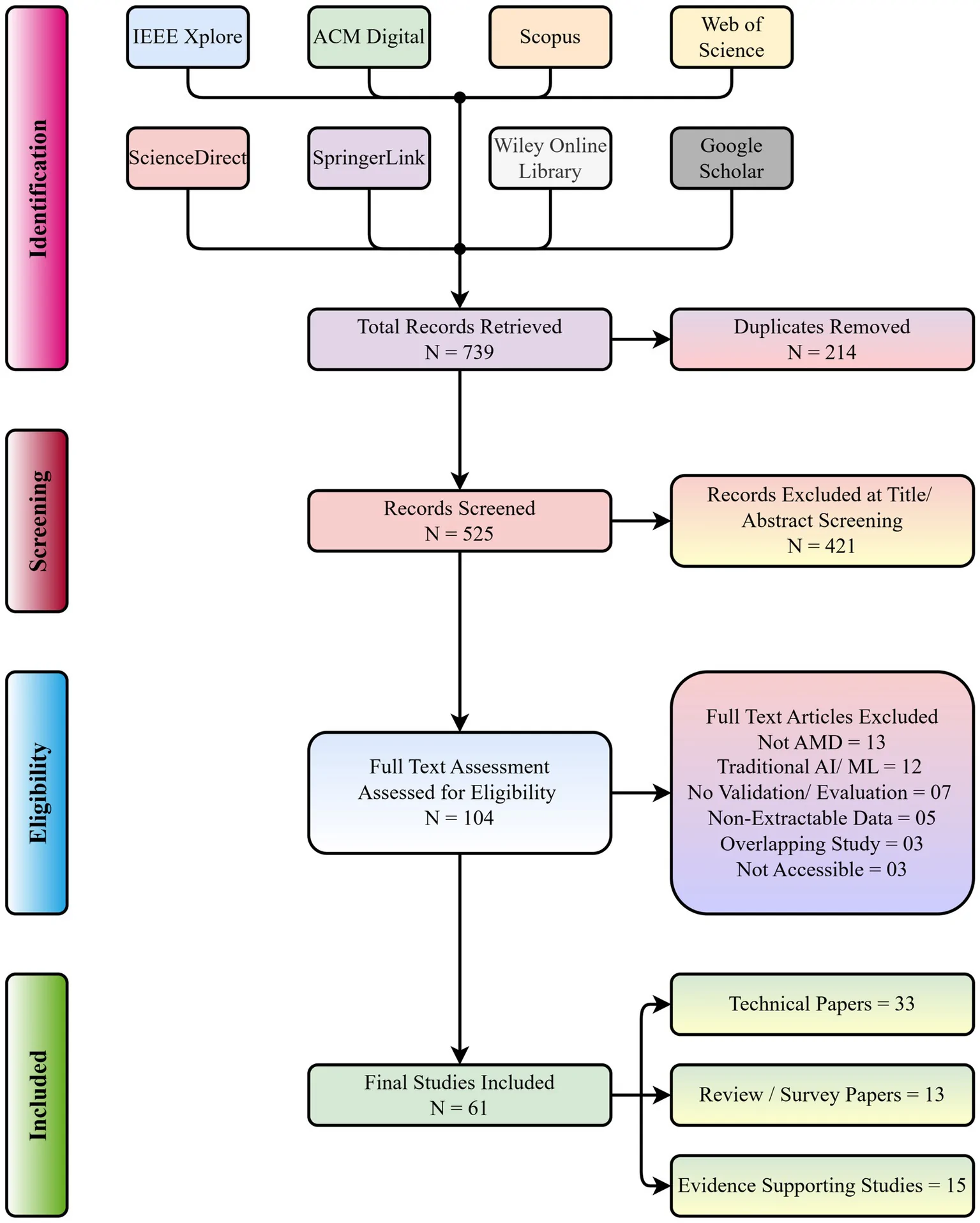
PRISMA-guided SLR diagram of the study.

**Table 3:** Inclusion and Exclusion criteria of the study for AMD.

No.	Inclusion criteria	Exclusion criteria
1	Study focuses on AMD, malware classification, malicious application, or malware-family analysis.	General malware focus and without Android-specific data, artifacts, or evaluation.
2	Study proposes, evaluates, compares, analyzes, or represents a DL method.	Focuses on traditional ML without DL and components.
3	Study uses Android-specific artifacts, data, samples, traffic, or malware family labels.	Purely conceptual with no empirical evaluation or evidence.
4	Study reports empirical evaluation using relevant metrics.	Does not disclose sufficient information about the dataset, validation, or protocol.
5	Study was published between 2021–2026 and was available in English.	Duplicate, abstract, blog, non-peer-reviewed, unavailable full text, or not in English.
6	Provides sufficient methodological rigor.	Lacks enough information to assess architecture, artifacts, validation, or evidence quality.

To further reduce selection bias, two reviewers screened and assessed eligibility independently. In the first stage, both reviewers checked the titles and abstracts against the inclusion and exclusion criteria. In the second stage, the same reviewers evaluated full-text articles for their relevance to Android, whether they involved DL-based detection or representation learning, the presence of empirical validation, and methodological completeness. Any disagreements were first settled through discussion by reexamining the study considering the predefined eligibility criteria; however, no disagreement was reached on the final inclusion. The same consensus process was applied during evidence coding to improve consistency in the classification of architecture, the mapping of artifacts, and the scoring of evidence credibility.

### 2.2 Coding

To strengthen credibility and assessment, a structured coding matrix was developed to ensure consistency for reviewed studies and their respective required characteristics. The coding process was organized around three analytical dimensions aligned with the RQs. The first dimension captured architecture evolution and addressed RQ1. The second dimension mapped DL architecture to Android artifacts and addressed RQ2. The third dimension assessed evidence credibility and addressed RQ3-RQ5. The data extraction and coding categories are mentioned in Table 4.

**Table 4:** Data extraction and coding matrix.

Coding category	Extracted information
Bibliographic information	Author, year, venue, article type
AMD paradigm	Static, dynamic, traffic-based, hybrid, image-based, sequence-based, graph-based
Android artifacts	Permissions, API calls, opcode sequences, bytecode, DEX files, network flows, runtime traces, malware images, graph structures
DL architecture family	CNN, RNN/GRU, autoencoder, GNN, Transformer, attention-based, hybrid, lightweight, XAI-oriented
Dataset information	Dataset name, sample size, benign/malware distribution, malware families or categories, sample source, sample age
Validation protocol	Train-test split, cross-validation, temporal split, family-aware split, cross-dataset testing, class imbalance handling
Performance metrics	Accuracy, precision, recall, F1-score, AUC, false positive rate, false negative rate, class-wise metrics
Robustness evidence	Obfuscation, evasion, perturbation, adversarial testing, stress testing
Explainability evidence	SHAP, LIME, Grad-CAM, attention visualization, artifact-level explanation
Reproducibility evidence	Code availability, data availability, preprocessing details, hyperparameters
Deployment evidence	Model size, parameter count, inference latency, memory usage, inference cost, real-device or edge feasibility

To put the evaluation-centered aim of this study into practice, an evidence-credibility scheme (presented in Table 5) was applied to the 28 non-redundant primary technical studies selected from 33 primary technical studies. The framework evaluates the extent to which each study report evidence supporting its AMD claims. It does not disqualify studies with lower scores. Instead, it interprets how strongly each study supports claims of reliability, robustness, explainability, reproducibility, and deployment feasibility. Each study was examined according to nine evidence dimensions. Each dimension was scored 0, 1, or 2. A score of 0 was given when the criterion was not reported or weakly reported, 1 when it was partially reported, and 2 when it was clearly reported. The maximum score for each study was 18. This scoring system is consistent with the evidence matrix reported in Section 3.

**Table 5:** Evidence-credibility scoring framework.

Criterion	Evaluation focus	Score 0	Score 1	Score 2
Dataset transparency	Dataset name, source, sample size, label basis, class distribution	Not reported	Partially reported	Clearly reported
Artifact/feature clarity	Android artifacts, preprocessing, feature extraction	Not reported	Partially described	Clearly described
Architecture clarity	Model structure, training setup, key hyperparameters	Not reported	Partially described	Clearly described
Metric adequacy	Metrics beyond accuracy, class-wise or security-relevant metrics	Accuracy only or unclear	Basic metrics reported	Multiple relevant metrics reported
Validation realism	Split strategy, temporal/family/cross-dataset awareness	Not reported or weak	Conventional split only	Realism-aware validation reported
Robustness/Evasion Evidence	Obfuscation, evasion, perturbation, adversarial, or stress testing	Not reported	Partially addressed	Clearly evaluated
XAI/Interpretability Evidence	SHAP, LIME, Grad-CAM, attention visualization, saliency maps, or artifact-grounded explanation	Not reported	Explanation reported but weakly grounded	Explanation mapped to Android artifacts
Reproducibility Support	Code, data access, preprocessing, hyperparameters	Not reported	Partially reproducible	Sufficiently reproducible
Deployment evidence	Model size, latency, memory, inference cost, device feasibility	Not reported	Partially reported	Clearly reported

Evidence level interpretation		
Score range	Evidence level	Description/Interpretation
0–7	Weak Evidence	Reported results have limited methodological support
8–13	Moderate Evidence	Results are partially supported, but an important evidence gap remains
14–18	Strong Evidence	Claims are supported by relatively complete methodological evidence

For architecture-level synthesis, 33 primary technical studies were retained, but the detailed evidence-strength matrix in Section 3 contains 28 non-redundant representative primary studies. The other five studies were retained in the qualitative synthesis but not tabulated separately because their architecture family, artifact type, or validation claim overlapped considerably with represented studies. This approach avoids overrepresentation of similar methodological designs, while maintaining the full qualitative coverage of the retained primary technical corpus.

### 2.3 Validation

The synthesis was performed in three steps. The first stage was the classification of architecture, where the studies were grouped according to the DL architecture family, such as CNNs, sequential models, autoencoders, GNNs, transformers, models based on attention, hybrid models, lightweight models, and models oriented to XAI. This stage answered RQ1 by identifying the evolution trends of DL architectures during 2021–2026. The second stage was artifact-to-architecture mapping, where each architecture family was mapped to the Android artifacts and feature representations they learn from. This stage enabled us to answer RQ2 with clarification on whether reported model performance was related to architectural innovation, feature representation, dataset properties, or validation design.

The third stage was the evidence-credibility synthesis. In this stage, the studies were interpreted based on the validity, validation realism, robustness, explainability, reproducibility, and deployment readiness of the dataset. This phase dealt with RQ3- RQ5. The synthesis did not rank models by reported accuracy because direct numerical comparison across studies is unreliable when datasets, feature spaces, malware families, validation protocols, and metrics vary. Instead, the synthesis looked at whether there was sufficient credible evidence to support claims of reliability, robustness, explainability, reproducibility, and deployment potential.

This study complements the systematic review with a controlled empirical validation using CICMalDroid 2020. The purpose of this validation is not to establish a new state-of-the-art Android malware detector, but to demonstrate how model interpretation changes when clean cross-validation is supplemented with class-wise reliability, malware false-negative behavior, repeated-run stability, statistical uncertainty, and feature-level robustness testing. The empirical task was treated as multi-class classification over the CICMalDroid 2020 categories used in the study, including benign application and malware categories such as adware, banking malware, SMS malware, and riskware. Representative DL architectures were selected according to the architecture families identified in the review. They include CNN, GRU, Transformer, and Hybrid CNN-LSTM models. The chosen models are for spatial feature learning, sequential modeling, contextual attention-based learning, and hybrid representation learning, respectively. The models were not used to exhaust all possible AMD architectures, but to support a controlled comparison of representative architecture families under the same reliability-audit protocol. The validation was structured around six evaluation perspectives, presented in Table 6.

**Table 6:** Empirical validation design.

No.	Evaluation view	Purpose	Main metrics
1	Dataset composition	Establishes class distribution and imbalance in CICMALDroid 2020	Class count, class percentage, imbalance pattern
2	Clean cross-validation	Establishes benchmark performance under clean input conditions	Accuracy, macro precision, macro recall, macro F1-score, fold-level results
3	Class-wise detection reliability	Examines category-level reliability across benign and malware classes	Class-wise F1-score, malware macro-recall, malware false-negative rate
4	Repeated-run stability	Assesses whether model performance is consistent across random seeds/runs (5)	Mean macro F1, standard deviation, confidence interval, repeated-run count
5	Statistical comparison	Test whether observed model differences are supported beyond descriptive ranking	Paired test or bootstrap confidence interval using fold-level/repeated-run macro F1-scores
6	Multi-level feature-masking robustness	Tests sensitivity to disturbed feature evidence at increasing perturbation levels	Clean F1, masked F1 at 5%, 10%, 20%, and 30%, robustness degradation rate, robustness retention score

All the models were implemented under the same experimental configuration to ensure a fair and controlled comparison. The idea was not to optimize each architecture individually but to compare representative DL architectures under the same constraints of pre-processing, splitting, training, and evaluation. The same CICMalDroid 2020 feature matrix was used in CNN, GRU, Transformer, and Hybrid CNN-LSTM models. The feature vectors were reshaped into architecture-compatible input formats as needed, without changing the underlying evidence source. To avoid information leakage, numeric features were normalized using Min–Max scaling fitted only on the training partition and then applied to the validation and test partitions. For each fold of the cross-validation, 20% of the training partition was held out as an internal validation subset for early stopping, while the test fold remained unseen for the final evaluation. Adam optimizer was used for all model training.

All models were trained using the Adam optimizer with cross-entropy loss, an initial learning rate of 0.001, a batch size of 32, and a maximum of 50 epochs. We used early stopping on validation loss with a patience of 10 epochs and saved the best validation checkpoint for testing. All models were trained using the same feature set, folding assignments, random-seed protocol, preprocessing steps, optimization settings, and stopping rules. Experiments were implemented using Python 3.12.13 running on a Linux 6.6.122 + x86_64 runtime in Google Colab with TensorFlow 2.20.0, scikit-learn 1.6.1, NumPy 2.0.2, and Pandas 2.2.2. The GPU runtime was an NVIDIA-Tesla T4 GPU with 15 GB of memory, NVIDIA driver 580.82.07, and CUDA 13.0. The outputs of the experiments were supplemented by the runtime type, GPU model, package versions, and random-seed settings to account for variability in Google Colab hardware allocation across sessions and to enable reproducibility.

The clean evaluation reported accuracy, macro precision, recall, and F1-score. Accuracy was retained for comparability with previous AMD studies, but macro-averaged metrics were emphasized because the CICMalDroid 2020 task class is imbalanced. Class-wise F-1 scores were further reported to identify category-specific weaknesses that can be hidden by aggregate scores. Since missed malware samples are security-critical and influence the reliability of AMD systems, macro-recall and false-negative rate were included. The repeated-run stability was tested by training each architecture over multiple independent runs with different random seeds but with the same preprocessing and data-splitting protocol. The repeated-run results were reported in terms of mean macro F1-score, standard deviation, and confidence interval-based uncertainty estimates. Statistical comparisons were made using macro F1-scores on repeated runs or fold-levels where pairwise claims for model superiority or stability were made. These tests were only used to support those differences that were statistically or practically distinguishable; otherwise, differences were interpreted descriptively and with caution. The robustness degradation rate and robustness retention score were computed as follows:


$$\text{Robustness Degradation Rate}\;(\%)=[\frac{\text{Clean}\;F1-\text{Perturbed}\;F1}{\text{Clean}\;F1}\;]\times 100$$

$$\text{Robustness Retention Score}=\frac{\text{Perturbed}\;F1}{\text{Clean}\;F1}$$

Feature-level robustness was assessed using multi-level test-time feature masking. The models were trained with clean training data, and the perturbation was applied only to the test set. Masking was applied to 5%, 10%, 20%, and 30% of non-label input features. For binary or one-hot encoded features, the selected feature values were set to 0. For normalized continuous features, selected values were replaced by the corresponding training set feature mean. Each masking level was rerun with different masking seeds and the macro F1-score obtained was reported separately for each perturbation level. To quantify the amount of clean performance lost under disturbed feature evidence, we computed the robustness degradation rate and robustness retention score. This feature-masking experiment should be understood as a controlled feature-stability test, not as a direct simulation of APK-level obfuscation, adversarial code rewriting, repackaging, reflection, dynamic payload loading, or sandbox evasion. It aims to study the stability of model rankings and reliability conclusions when part of the input evidence is perturbed. The interpretation stage combined the review and empirical validation: The architecture synthesis and artifact-to-architecture mapping address RQ1 and RQ2, and the evidence-credibility framework and controlled empirical validation jointly answer RQ3–RQ5.

## 3 Architecture-centered results and evidence classification

This section reports on the architecture-centered findings of the retained study base. It addresses RQ1 and RQ2 by classifying DL-based AMD architectures and mapping them to the Android artifacts from which they learn. It also creates the foundations for RQ3-RQ5 by evaluating whether the architectural claims are supported by sufficient evidence. The significant finding revolves around the fact that DL-based AMD has become architecturally diverse, but evidence maturity remains insignificant. All investigated methods do demonstrate methodological expansion, but it cannot be considered as progress unless results are credible and validated with evidence.

### 3.1 Landscape

As described in Section 2, the architecture-level synthesis comprises 33 primary technical studies for architecture-artifact evidence. Therefore, this subsection reports the architecture landscape that emerges from the primary technical corpus. The evaluated evidence clearly indicates the shift from manually engineered static features toward learned and transformed representations. Numerous studies have turned Android artifacts into image-like structures to leverage mature computer-vision architectures. For instance, RT-Droid (Tasyurek and Arslan, 2023) converts Android manifest permission vectors into small RGB QR-style images. Later, it reports on near real-time analysis with YOLOv5 at 0.019 s/application. This shows how representation design can directly influence the deployment feasibility.

Similarly, the TCN bytecode-image study (Zhang W. et al., 2021) also transforms androidmanifest.xml and DEC data-section information into grayscale bytecode images but learns sequence-like patterns from transformed static artifacts via temporal convolution. These cases show that progress is not only architectural but also representational. But researchers and scientists should not take this shift as automatic methodological maturity. The transformation of permissions, bytecode, DEX files, or traffic into images can improve the compatibility of the model. It can also introduce semantic loss, preprocessing dependency, benchmark sensitivity, and new deployment costs. Thus, the central question is not whether transformed representations improve reported accuracy, but whether they preserve meaningful malware evidence and remain reliable under realistic evaluation constraints. The distribution of primary technical studies by architecture is presented in Table 7.

**Table 7:** Distribution of architecture families and design orientations across primary AMD studies.

Architecture/Design family	No. of studies	Percentage	Main evidence source	Main observation
CNN /image-based models	17	51.5%	DEX images, bytecode images, opcode images, manifest features, permissions, malware visual representations	Dominant representation-learning direction; widely used for scalable static screening, but often sensitive to image transformation, padding, and obfuscation.
Sequential models	8	24.2%	API-call sequences, opcode sequences, system-call traces, behavior logs, traffic/event sequences	Useful for behavioral and temporal evidence, especially when execution order matters; however, performance depends on sequence completeness and observation quality.
Transformer/attention models	7	21.2%	Tokenized API/opcode sequences, traffic tokens, DEX/image tokens, self-attention feature maps	Emerging contextual-representation direction; improves long-range dependency modeling but often requires higher computation and stronger validation.
GNN/graph-based models	2	6.1%	Function-call graphs, API dependency graphs, control-flow or program-structure graphs	Structurally rich and semantically meaningful, but highly dependent on reliable graph extraction and vulnerable to reflection, dynamic loading, or unresolved calls.
Autoencoder/latent-representation models	3	9.1%	Bytecode vectors, image features, tabular static features, behavioral representations	Useful for compression, anomaly-oriented learning, and representation refinement, but often weaker in interpretability and deployment evidence.
Hybrid/multimodal models	11	33.3%	Static + dynamic features, image + sequence features, CNN–RNN fusion, ensemble learning, traffic + API evidence	Integrates multiple evidence sources and often improves aggregate performance but increases pipeline complexity and reproducibility burden.
Lightweight/deployment-oriented models	6	18.2%	Compact CNNs, transfer CNNs, distilled models, efficient image/static features, lightweight classifiers	Promising for operational feasibility, but many studies still report limited evidence on latency, memory, energy cost, and real-device constraints.
XAI-oriented models	6	18.2%	APIs, permissions, DEX regions, image saliency, SHAP/LIME explanations, code-localization outputs	Important emerging direction for analyst trust, but explanations remain uneven and are not always mapped to verifiable Android artifacts.

### 3.2 Mapping

Building on the distribution reported in Table 7, this subsection maps DL architecture families to the Android artifacts from which they learn. Figure 2 presents the architecture classification developed from the reviewed studies. This links four layers, i.e., analysis modality, Android artifact or feature representation, DL, architecture family, and evidence/deployment concern. This classification is architecture-centered but not isolated from the architecture. It does not consider the methods (CNNs, GRU, transformers, hybrid ensembles) as self-contained categories. Rather, each model family is characterized by the Android evidence source on which it trains. This distinction is critical, as a CNN trained on DEX images, a YOLO trained on permission QR-code-like images, a BiGRU trained on HTTP/TCP flows, and a transformer trained on traffic tokens. This represents different detection assumptions and failure modes. Thus, a direct comparison using reported accuracy is methodologically weak unless the representation dataset and validation context are taken into account.

**Figure 2:**
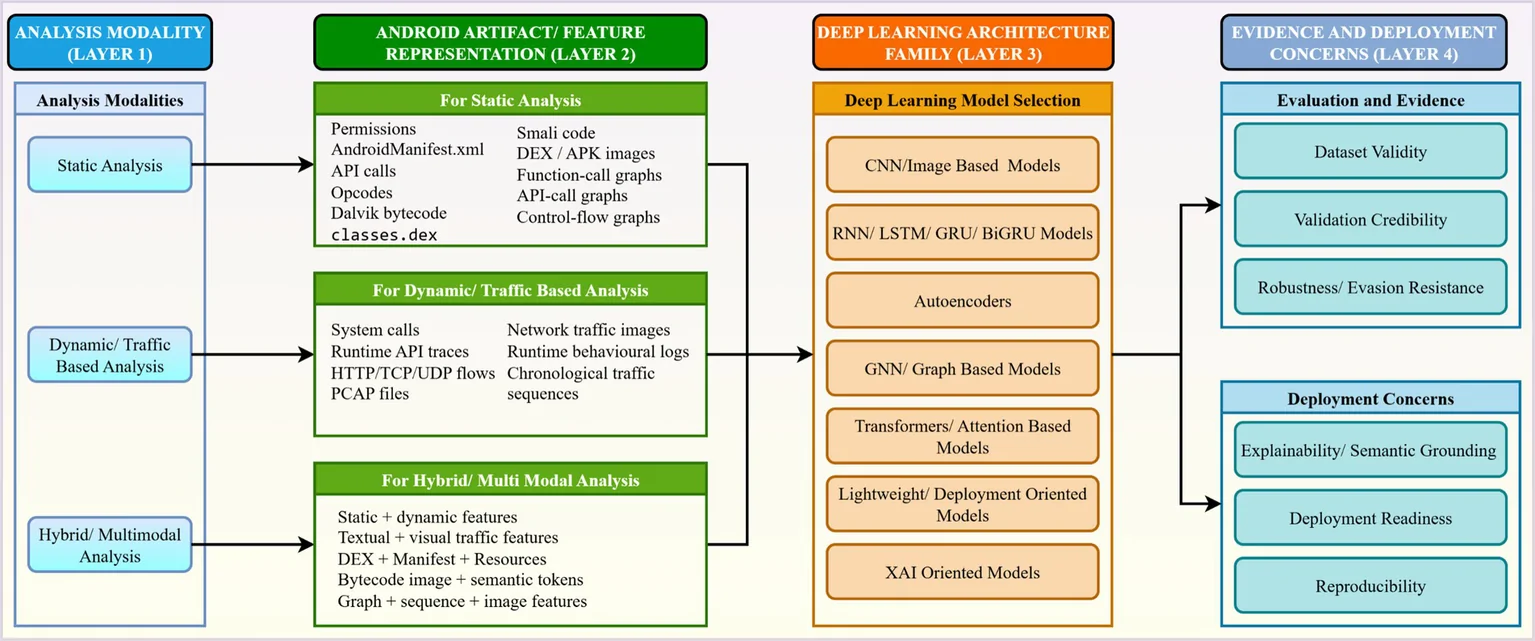
Classification or taxonomy of DL Based AMD.

The taxonomy brings out three important patterns. The first is CNN-based image learning, which is one of the most mature and widely used directions. In this approach, Android artifacts, including DEX files, bytecode, permissions, opcode transitions, manifest information, and resources, are mapped into grayscale, RGB, Markov, QR-like, or multi-channel images, and then provided to CNN or transfer-learning architectures. This family of architectures is attractive because it allows existing computer vision models to be reused to classify Android malware. However, its main limitation is the semantic gap between visual separability and real malicious behavior (Vu and Jung, 2021; Maganur et al., 2025; Papankar and Mandaogade, 2024). The model can learn discriminative image patterns without necessarily learning malware semantics. Thus, image-based AMD evidence is strongest when the visual representation links to meaningful Android artifacts such as DEX regions, bytecode segments, permissions, opcode structures, or manifest elements. Designs for representation, like in RT-Droid (Tasyurek and Arslan, 2023) and the TCN bytecode-image approach (Zhang W. et al., 2021), show how representation design can contribute to detection efficiency, but also why image transformation, padding, preprocessing, and semantic preservation must be considered when interpreting reported performance.

Second, sequential, graph-based, and Transformer/attention-based models are increasingly used to capture behavioral order, structural dependency, and long-range contextual relations. Sequential models are used for processing API calls, system calls, opcode sequences, and network flows. Graph models are trained on function-call graphs, API graphs, or control-flow graphs. Transformer and attention-based models learn the context relations from tokenized representations of traffic, opcode, API, or DEX. These models are conceptually more powerful for complex malware behavior, but their credibility is contingent on trace completeness, reliability of graph extraction, interpretability of attention, and realism of validation (Zhang N. et al., 2021; Chen et al., 2026; Sasidharan and Thomas, 2021; Almakayeel, 2024; Shakib, 2025). Sequential evidence is based on the observation of relevant behavior. The dependability of graph evidence depends on dependable graph extraction, which can be affected by reflection, dynamic loading, obfuscation or unresolved calls. The attention-based evidence depends on whether weights or highlighted regions are mapped back to profound Android artifacts by default, instead of being treated as explanations.

Third, hybrid, lightweight, and XAI-oriented models show that the field is maturing toward operational feasibility and analyst trust, rather than classification accuracy alone. Hybrid models attempt to combine multiple sources of evidence, lightweight models focus on memory and latency constraints, and XAI-oriented models aim to make the model outputs interpretable. However, these directions need to be more supported by stronger evidence. Hybrid models need ablation studies, missing-modality tests, and component-level analysis to show which evidence source drives the reported gain. Lightweight models need latency, memory, hardware, and real-device reporting. XAI-oriented models need explanations that are mapped to verifiable Android artifacts rather than generic feature importance or visual heatmaps (Bashir et al., 2024; Yao et al., 2025; Ma et al., 2024; El Youssofi and Chougdali, 2025). The artifact-to-architecture mapping is summarized in Table 8. The table should be interpreted as an evidence map, not as a ranking of model families.

**Table 8:** Artifact-to-architecture mapping in reviewed DL-based AMD studies.

Android evidence/Representation	Common architecture families	Representative use	Main reliability concern
Permissions and manifest features	CNN, YOLO-style CNN, lightweight CNN, ML/DL hybrids (Tasyurek and Arslan, 2023; Zhang W. et al., 2021; Ma et al., 2024; Wu et al., 2023)	Fast static screening, permission-image or QR-style encodings	Semantically shallow evidence; vulnerable to permission manipulation and benign-looking padding
DEX, bytecode, opcode, and APK images	CNN, transfer CNN, ViT, TCN, autoencoder, hybrid models (Jo et al., 2023; Zhang W. et al., 2021; Bakır and Bakır, 2023; Yapici, 2025; Xing et al., 2022; Yadav et al., 2022; Bakour and Ünver, 2021; Bakır, 2025)	Image-based malware classification using grayscale, RGB, Markov, or multi-channel representations	Semantic gap between visual patterns and malware behavior; sensitivity to transformation and preprocessing
API calls, system calls, and opcode sequences	LSTM, GRU, BiGRU, Transformer, attention models (Qiu et al., 2020; Zhang N. et al., 2021; Chen et al., 2026; Sasidharan and Thomas, 2021; Almakayeel, 2024; Shakib, 2025; Amer and El-Sappagh, 2022)	Behavioral or temporal detection where order and context matter	Sequence incompleteness, trigger dependency, truncation, and observation-window bias
HTTP/TCP flows and network traces	CNN–BiGRU, BERT/Transformer, hybrid traffic models (Ullah et al., 2024; Ullah et al., 2022a; Shen et al., 2023; Ullah et al., 2022b)	Network-behavior detection using textual, sequential, or visual traffic representations	Dependence on traffic capture quality, sandbox triggering, encryption, privacy, and runtime conditions
Function-call, API, and control-flow graphs	GNN, GCN, graph-attention models (Cai et al., 2021)	Structural learning from program dependency or call relationships	Fragile graph extraction under obfuscation, reflection, dynamic loading, or unresolved calls
Multimodal static–dynamic evidence	Hybrid, ensemble, multimodal DL, transfer-learning models (Shen et al., 2023; Ullah et al., 2022b; Alfaw et al., 2025; Kardgar et al., 2026)	Fusion of static artifacts, traffic traces, image features, and behavioral sequences	Attribution difficulty; requires ablation, missing-modality testing, and reproducibility support
Model explanations and artifact localization	XAI-oriented CNN, Transformer, hybrid models (Ullah et al., 2024; Ullah et al., 2022a; Jo et al., 2023; El Youssofi and Chougdali, 2025; Baghirov, 2025)	Grad-CAM, SHAP, LIME, attention maps, DEX/method-level localization	Explanation quality depends on whether outputs are mapped to meaningful Android artifacts
Compact or deployment-oriented representations	Lightweight CNN, distilled models, efficient transfer models (Tasyurek and Arslan, 2023; Yao et al., 2025; Ma et al., 2024; Alfaw et al., 2025)	Edge or real-time AMD with reduced model size or inference cost	Deployment claims require latency, memory, energy, hardware, and real-device evidence

Static analysis is persistently dominant due to fast pre-execution screening and large-scale app assessment. Thus, the popularity of CNN-based static analysis can be explained. But the prevalence of static analysis reflects operational convenience as well as detection reliability. Static artifacts can be obfuscated, reflected, repacked, dynamically loaded, or inserted with benign code. High static-model accuracy does not imply robustness against adaptive AMD. This trade-off is clearly articulated by image-based static studies. They often report high performance by converting APK components into grayscale, RGB, Markov, or multi-channel images. However, the security implications of visual patterns are not always obvious. A structural regularity might be encoded by a bytecode image, but the model may also learn dataset specifics (such as byte distributions, family duplication, packaging artifacts, or compiler traces). This is the significance of studies such as the Vision Transformer-based detector. Rather than stopping at image-level classification, it attempts to map the attention region back to the DEX offsets, class names, and method names. This partially narrows the semantic gap between visual features and Android program logic (Jo et al., 2023).

Dynamic and traffic-based studies are more behaviorally meaningful as they capture event sequences, communication patterns, and runtime activity. For instance, NMAL-Droid (Ullah et al., 2024) extracts HTTP traces and TCP flows, creates word2vec-based embeddings, and combines CNN feature learning with BiGRU sequence modeling. Also, transformer-based network malware detection (Ullah et al., 2022a) is performed using BERT-like contextual representation over HTTP/TCP flows and fusion of textual and visual traffic features. Methodologically, these studies are important because they bring AMD closer to observed behavior. The limitation includes the behavior needing to be initiated and recorded. If the malware detects the sandbox environment, delays execution, or requires specific user or network conditions, the resulting trace may be incomplete.

Hybrid and multimodal approaches attempt to overcome the weakness of single sources by fusing static artifacts, traffic traces, textual features, visual representations, or sequential behavior. Conceptually, this is appealing because AM is seldom represented by a single signal. This shows how different sources of malware evidence can complement each other through hybrid transfer-learning studies combining HTTP/TCP textual features with PCAP-derived visual texture features (Ullah et al., 2022b). Yet, hybrid models complicate the attribution of evidence as well. Without ablation analysis, missing modality tests, and robustness evaluation, it is difficult to attribute performance gains to architecture design, feature fusion, ensemble averaging, class balancing, or dataset-specific effects.

Similarly, the cautions are broadly applied to feature representation as well. Though the permissions and manifest features are efficient, they are semantically shallow. Furthermore, it is evident that bytecode and DEX representations are richer, but they are vulnerable to transformation. The CNNs are effective with image-based features, but they may also introduce semantic gaps. Although sequential traces capture the behavior, they are still dependent on execution coverage. On the other hand, the graph representation preserves the structure, but it relies on fragile extraction. Likewise, communication behavior is revealed by network features, but it raises concerns about capture and privacy. Therefore, Shar et al. (2023) empirically evaluated the static, dynamic, and combined features under the settings of time and space aware. It shows that the performance interpretation depends strongly on feature type and validation design. Hence, the representational diversity of the field is both a strength and a source of methodological uncertainty.

Thus, the main finding of this descriptive synthesis is one of caution rather than celebration. AMD is becoming more technically rich, but the architectural diversity is outpacing evidence maturity. Many studies propose more sophisticated models but rely on aging datasets, random splits, limited cross-dataset validation, and weak robustness testing. This also includes incomplete deployment reporting or semantically unvalidated explanations. Therefore, the next subsection shifts from architecture-artifact mapping to evidence assessment. It evaluates whether the claims associated with each architecture family are supported by evidence.

### 3.3 Evidence

In this subsection, we rely on the artifact-to-architecture mapping of Section 3.2 to evaluate whether the claims associated with recent DL-based AMD architectures are supported by mature empirical evidence. The aim is not to rank studies according to reported accuracy, but to determine whether each study provides sufficient evidence of reliability, robustness, explainability, reproducibility, and deployment-oriented interpretability. The nine-dimension evidence-credibility framework defined in Section 2.2 is operationalized across 28 non-redundant representative studies in Table 9. These studies were selected as they most directly represent the architecture families, Android artifact types, and validation claims investigated in this review.

**Table 9:** Evidence strength matrix of 28 representative evidence coded primary technical studies.

Ref.	Architecture/Model family	Main evidence	Strengths											Remarks
			DT	AF	AR	MA	VR	R/E	X/I	RP	DE	SR	EL	
Tasyurek and Arslan (2023)	YOLOv5/Transfer CNN	Permission QR code-like images	**✓**	**✓**	**✓**	**✓**	**✓**	**x**	**x**	**◐**	**✓**	13	M	The representation may be semantically shallow but shows strong real-time evidence.
Ullah et al. (2024)	CNN-BiGRU Ensemble + XAI	HTTP Traces/TCP Flows	**✓**	**✓**	**✓**	**✓**	**◐**	**◐**	**✓**	**◐**	**◐**	14	S	Its temporal generalization remains uncertain, but it shows strong network traffic and explainability evidence.
Ullah et al. (2022a)	BERT + CNN + Ensemble +XAI	HTTP/TCP flows + visual texture features	**✓**	**✓**	**✓**	**✓**	**◐**	**◐**	**✓**	**◐**	**◐**	14	S	Shows very high computational complexity but shows important transformer/XAI evidence.
Jo et al. (2023)	Vision Transformer/XAI	DEX RGB images	**✓**	**✓**	**✓**	**✓**	**✓**	**◐**	**✓**	**◐**	**◐**	15	S	Since attention is mapped back to DEX artifacts, it shows strong transformer/XAI evidence.
Shar et al. (2023)	RNN/LSTM Benchmark	Static/dynamic feature sets	**✓**	**✓**	**✓**	**✓**	**✓**	**◐**	**x**	**◐**	**◐**	13	M	Since it evaluates time- and space-aware bias, it seems methodologically important.
Zhang W. et al. (2021)	TCN/Bytecode-image Sequence Modeling	Manifest + DEX data-section images	**✓**	**✓**	**✓**	**✓**	**◐**	**x**	**x**	**◐**	**◐**	11	M	The random splits limit the practical inference; however, it shows useful temporal convolutional evidence.
Ullah et al. (2022b)	CNN + Voting Ensemble	HTTP/TCP text + PCAP images	**✓**	**✓**	**✓**	**✓**	**◐**	**◐**	**◐**	**◐**	**◐**	13	M	The pipeline is complex, and component contributions need ablation, but it is useful hybrid evidence.
Bakır and Bakır (2023)	Autoencoder/Representation Learning	classes.dex image features	**✓**	**✓**	**◐**	**✓**	**◐**	**x**	**◐**	**◐**	**x**	10	M	Have very limited robustness and deployment evidence but are useful for latent feature extraction
Ravi et al. (2022)	Attention/Multiview DL	Image-based malware views	**◐**	**◐**	**✓**	**✓**	**◐**	**x**	**◐**	**x**	**x**	8	M	Has partial Android scope, but relevant for attention-based learning.
Amer and El-Sappagh (2022)	LSTM/Sequence Learning	API calls, system calls, permissions	**✓**	**✓**	**✓**	**✓**	**✓**	**◐**	**x**	**◐**	**◐**	13	M	Shows strong behavioral sequence and evidence with values of early warnings.
Wu et al. (2023)	RL Assisted Feature Selection	Permission, intents, opcodes	**✓**	**✓**	**◐**	**✓**	**◐**	**x**	**x**	**◐**	**◐**	10	M	Shows not an end-to-end DL detection but shows significance for feature-reduction efficiency.
Cai et al. (2021)	GCN/Graph Learning	Function call graphs	**✓**	**✓**	**✓**	**✓**	**◐**	**◐**	**◐**	**◐**	**x**	12	M	It has a limitation in graph-extraction cost but shows strong evidence of structural learning.
Yapici (2025)	CNN/mage Based DL	Dalvik bytecode RGB/grayscale images	**✓**	**✓**	**✓**	**✓**	**✓**	**◐**	**x**	**◐**	**◐**	13	M	It has very limited real deployment testing but shows strong image-based evidence with dataset cleaning.
Ma et al. (2024)	Lightweight CNN/ShuffleNetV2-ECA	XML and DEX image features	**✓**	**✓**	**✓**	**✓**	**◐**	**x**	**x**	**◐**	**✓**	12	M	It shows limited generalization evidence but strong lightweight detection.
Vasan et al. (2024)	Broad learning/Image representation	Grayscale malware images	**◐**	**◐**	**✓**	**✓**	**◐**	**◐**	**x**	**◐**	**✓**	11	M	No exclusive Android evidence, but shows an efficient GPU approach.
Nethala et al. (2025)	Attention/Ensemble CNN	APK byte images	**◐**	**✓**	**✓**	**✓**	**◐**	**x**	**◐**	**x**	**x**	9	M	Performance reporting should be cautious but shows useful ensemble evidence.
Yao et al. (2025)	Lightweight CNN/Knowledge Distillation	Opcode Markov images	**✓**	**✓**	**✓**	**✓**	**✓**	**◐**	**x**	**◐**	**✓**	14	S	The generalization gap remains visible on new data, though it shows strong lightweight evidence.
Xing et al. (2022)	Autoencoder	Bytecode grayscale images	**◐**	**✓**	**◐**	**✓**	**◐**	**x**	**◐**	**x**	**x**	8	M	The semantic interpretability is weak but is useful for representation compression.
Yadav et al. (2022)	Efficient Net B0 + Stacking	DEX RGB images	**◐**	**✓**	**✓**	**✓**	**◐**	**x**	**x**	**◐**	**◐**	10	M	It is limited to robustness and temporal testing but shows strong transfer learning performance.
Mercaldo et al. (2024)	DCGAN/Adversarial Generation	Static/dynamic malware images	**◐**	**◐**	**✓**	**✓**	**◐**	**✓**	**x**	**◐**	**x**	10	M	It is not a conventional detection but is significant in adversarial/generative discussion.
Alfaw et al. (2025)	CNN, RNN, LSTM, CNN-LSTM	Static/dynamic indicators	**✓**	**✓**	**✓**	**✓**	**✓**	**◐**	**x**	**◐**	**✓**	14	S	It shows strong and deployment-oriented evidence. It includes mobile/TFLite-style feasibility.
El Youssofi and Chougdali (2025)	YOLO11/Image based XAI	DEX, Manifest, Resources RGB channels	**✓**	**✓**	**✓**	**✓**	**✓**	**◐**	**✓**	**◐**	**◐**	15	S	Needs a cautious interpretation but still shows very high accuracy, strong XAI, and cross-corpus evidence.
Bakour and Ünver (2021)	1D-CNN/image-feature hybrid	DEX, Manifest, Resources images	**✓**	**✓**	**✓**	**✓**	**◐**	**x**	**x**	**◐**	**◐**	11	M	Relies on older malware corpora but still shows important early image-based evidence.
Bakır (2025)	Transfer CNN/Bayesian Optimization	DEX Code images	**✓**	**✓**	**✓**	**✓**	**◐**	**x**	**x**	**◐**	**◐**	11	M	Limited robustness evidence but useful for optimization and transfer learning.
Laudanna et al. (2025)	Adversarial DL	Static feature vectors	**✓**	**✓**	**✓**	**✓**	**✓**	**✓**	**x**	**◐**	**x**	13	M	It shows important evidence for invasion risk but not for detection deployment.
Shen et al. (2023)	CNN-LSTM + Self-Attention	HTTP/TCP/UDP traffic images	**✓**	**✓**	**✓**	**✓**	**◐**	**◐**	**◐**	**◐**	**◐**	13	M	It shows dependency on traffic capture quality but also shows strong dynamic/traffic evidence.
Kardgar et al. (2026)	Sparse autoencoder + ML Classifier	Network/behavioral features	**✓**	**✓**	**◐**	**✓**	**◐**	**x**	**◐**	**◐**	**◐**	11	M	The final classifier of the study is not purely DL; however, it does have useful latent-feature compression
Baghirov (2025)	Light GBM + SHAP/LIME	CICMAL Droid tabular features	**✓**	**✓**	**◐**	**✓**	**◐**	**◐**	**✓**	**◐**	**x**	12	M	Not a core DL architecture, but it does show supporting XAI/methodological study and is useful for trustworthiness discussion.

Symbol	Meaning	Score
**✓**	Clearly Reported/Strong Evidence	2
**◐**	Partially Reported/Limited Evidence	1
**X**	Not Reported, Unclear, or Weakly Supported	0

Score range (SR)	Evidence level (EL)	Interpretation
0–7	Weak (W)	The study provides limited methodological support for its claims.
8–13	Moderate (M)	The study provides partial evidence, but important gaps remain.
14–18	Strong (S)	The study provides comparatively complete methodological support.

DT, Dataset Transparency; AF, Artifact/Feature Clarity; AR, Architecture Clarity; MA, Metric Adequacy; VR, Validation Realism; R/E, Robustness/Evasion Evidence; X/I; XAI/Interpretability Evidence; RP, Reproducibility Support; DE, Deployment Evidence.

The evidence-strength matrix illustrates that architectural innovation is often ahead of evidence reporting. Many papers give enough details about the datasets, Android artifacts, model architectures, and traditional performance measures. Still, fewer studies provide strong evidence for validation realism, robustness or evasion testing, artifact-grounded XAI, reproducibility, or deployment feasibility. Thus, studies with the highest reported accuracy are not necessarily the strongest studies in terms of the maturity of evidence. The strongest studies combine transparent datasets, well-defined Android artifacts, clear reporting of the architecture, adequate metrics, realistic validation design, and at least some evidence of robustness, interpretability, reproducibility, or deployment feasibility.

The matrix suggests that studies such as Ullah et al. (2024), Ullah et al. (2022a), Jo et al. (2023), Yao et al. (2025), El Youssofi and Chougdali (2025), Alfaw et al. (2025) offer comparatively stronger evidence as they provide explicit architecture and artifact descriptions coupled with stronger validation, XAI, robustness, or deployment-related support. But even studies that make the grade have limitations. For example, attention-based and XAI-oriented studies are more convincing if explanations can be mapped back to Android artifacts, e.g., localization at the DEX-region or method level (Jo et al., 2023). However, attention or saliency alone should not be interpreted as a causal explanation. Likewise, lightweight or real-time oriented studies offer useful deployment direction, but their claims are strongest only when latency, memory, hardware setting, and real-device feasibility are reported. Moderately scoring studies are still useful for architecture and representation synthesis, but their claims should be taken with a grain of salt when evidence of robustness, reproducibility, or deployment is lacking.

The aggregate evidence pattern is shown in Table 10. The table shows that the field is not deficient in basic experimental reporting. Dataset transparency, artifact/feature clarity, architecture clarity, and metric adequacy are generally well represented. The biggest weaknesses are those most directly related to real-world reliability: validation realism, robustness/evasion proof, XAI grounding, reproducibility support, deployment readiness.

**Table 10:** Aggregate evidence pattern across the 28 representative evidence-coded studies.

Evidence dimension	Clear evidence	Partial evidence	Weak/Not reported	Interpretation
Dataset transparency	22/28 (78.6%)	6/28 (21.4%)	0/28 (0.0%)	Dataset reporting is generally present.
Artifact/feature clarity	25/28 (89.3%)	3/28 (10.7%)	0/28 (0.0%)	Most studies describe their Android evidence source.
Architecture clarity	23/28 (82.1%)	5/28 (17.9%)	0/28 (0.0%)	Model families are usually identifiable.
Metric adequacy	28/28 (100.0%)	0/28 (0.0%)	0/28 (0.0%)	Standard performance metrics are consistently reported.
Validation realism	9/28 (32.1%)	19/28 (67.9%)	0/28 (0.0%)	Validation is usually present but often not realism-aware.
Robustness/evasion evidence	2/28 (7.1%)	14/28 (50.0%)	12/28 (42.9%)	Robustness remains a major evidence gap.
XAI/interpretability evidence	5/28 (17.9%)	8/28 (28.6%)	15/28 (53.6%)	XAI is emerging but still inconsistent.
Reproducibility support	0/28 (0.0%)	25/28 (89.3%)	3/28 (10.7%)	Reproducibility is mostly partial, not complete.
Deployment evidence	5/28 (17.9%)	15/28 (53.6%)	8/28 (28.6%)	Deployment evidence is visible but rarely complete.

Percentages in Table 10 are calculated using the 28 representative evidence-coded studies in Table 9 as the denominator. The broader architecture-family distribution reported in Table 7 is based on all 33 primary technical studies retained for architecture-level synthesis.

The aggregate pattern suggests that current DL-based AMD research is architecture-rich but evidence-incomplete. Metric adequacy is consistently reported, and the majority of studies clearly describe their dataset, sources of Android evidence, and model families. But only a few show clear evidence of realism-aware validation, robustness testing, artifact-grounded interpretability, or deployment feasibility. Reproducibility is also mostly partial rather than complete, with many studies providing some implementation detail but not enough to fully reproduce preprocessing, training, splits, hyperparameters, and evaluation conditions.

This finding supports RQ3 and partially supports RQ4 and RQ5. It shows that architecture-level progress cannot be assessed by accuracy alone since reported performance depends on the maturity of the surrounding evidence. CNN/image-based models are common and operationally attractive, but their reliability depends on semantic grounding and robustness to representation shifts. Sequential and traffic-based models can account for behavioral evidence, but their validity depends on the coverage of execution and the completeness of the traces. Transformer and attention-based models improve contextual learning, but their interpretability depends on mapping attention evidence to Android artifacts. Hybrid models use multiple signals, but it is hard to understand their contribution without ablation and missing-modality tests. While lighter models support deployment-oriented AMD, there is no clear real-world utility without full latency, memory, hardware, and real device evidence.

Section 3 shows that recent DL-based AMD has grown significantly in architecture and representation, but the foundation of evidence for reliability is still uneven. This further motivates conducting a controlled empirical validation in Section 4, where representative CNN, GRU, Transformer, and Hybrid CNN-LSTM models are evaluated on CICMalDroid 2020 using clean cross-validation, class-wise detection, malware false-negative behavior, repeated-run stability, and feature-masking robustness.

## 4 Empirical validation

This section provides a controlled empirical validation with CICMalDroid 2020 following the architecture-centric synthesis in Section 3. This validation aimed to empirically support the review’s main argument that “clean benchmark performance is not sufficient to judge the reliability of DL-based AMD”. The empirical validation is based on four representative architecture families: CNN, GRU, Transformer, and Hybrid CNN-LSTM. These models align with the major architectural trends pointed out in Section 3 (i.e., spatial/image-based learning, sequential modeling, contextual and hybrid representation learning). The evaluation focuses on dataset composition, clean cross-validation performance, class-wise malware reliability, malware false-negative behavior, repeated-run stability, and multi-level feature-masking robustness.

### 4.1 Dataset and clean performance

Figure 3 shows the composition of the CICMalDroid-2020 dataset used for empirical validation. The dataset is modeled as a multi-class classification problem with benign apps and malware families such as adware, banking malware, SMS malware, and riskware. The distribution of categories implies that the validation setting is not a straightforward balanced binary classification task. This requires class-wise evaluation and macro-averaged metrics, as aggregate accuracy can hide bad performance in less popular or more difficult malware classes.

**Figure 3:**
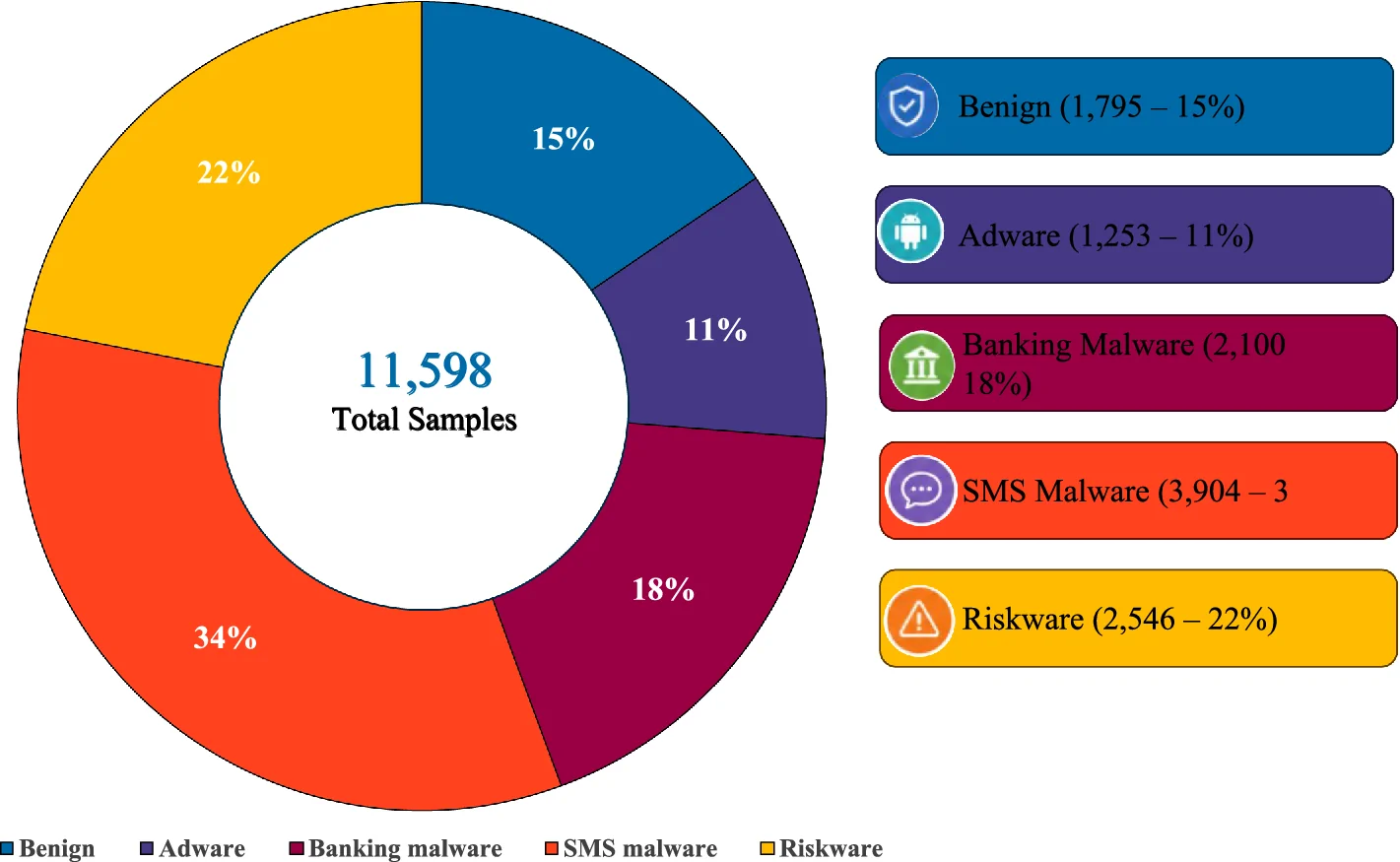
CICMalDroid 2020 dataset composition is considered for empirical validation.

Clean-cross validation performance is reported in Figure 4. All four models achieved competitive performance on the clean benchmark, with macro F1-scores between 0.895 and 0.955. The Hybrid CNN-LSTM achieved the best clean-performance profile under the shared evaluation protocol with an accuracy of 0.945 ± 0.004 and macro F1-score of 0.955. The accuracy and macro F1-score of the Transformer were 0.928 ± 0.006 and 0.925, respectively, while the GRU and CNN had an accuracy and macro F1-score of 0.907 ± 0.008 with macro F1 0.910 and 0.911 ± 0.005, macro F1 0.895, respectively. These results suggest that hybrid spatial-sequential learning and attention-based contextual representation can provide stronger clean benchmark performance than the CNN and GRU baselines in this controlled setting. Clean cross-validation, however, is understood to be only the baseline evidence (not the evidence) of robustness, operational reliability or deployment readiness.

**Figure 4:**
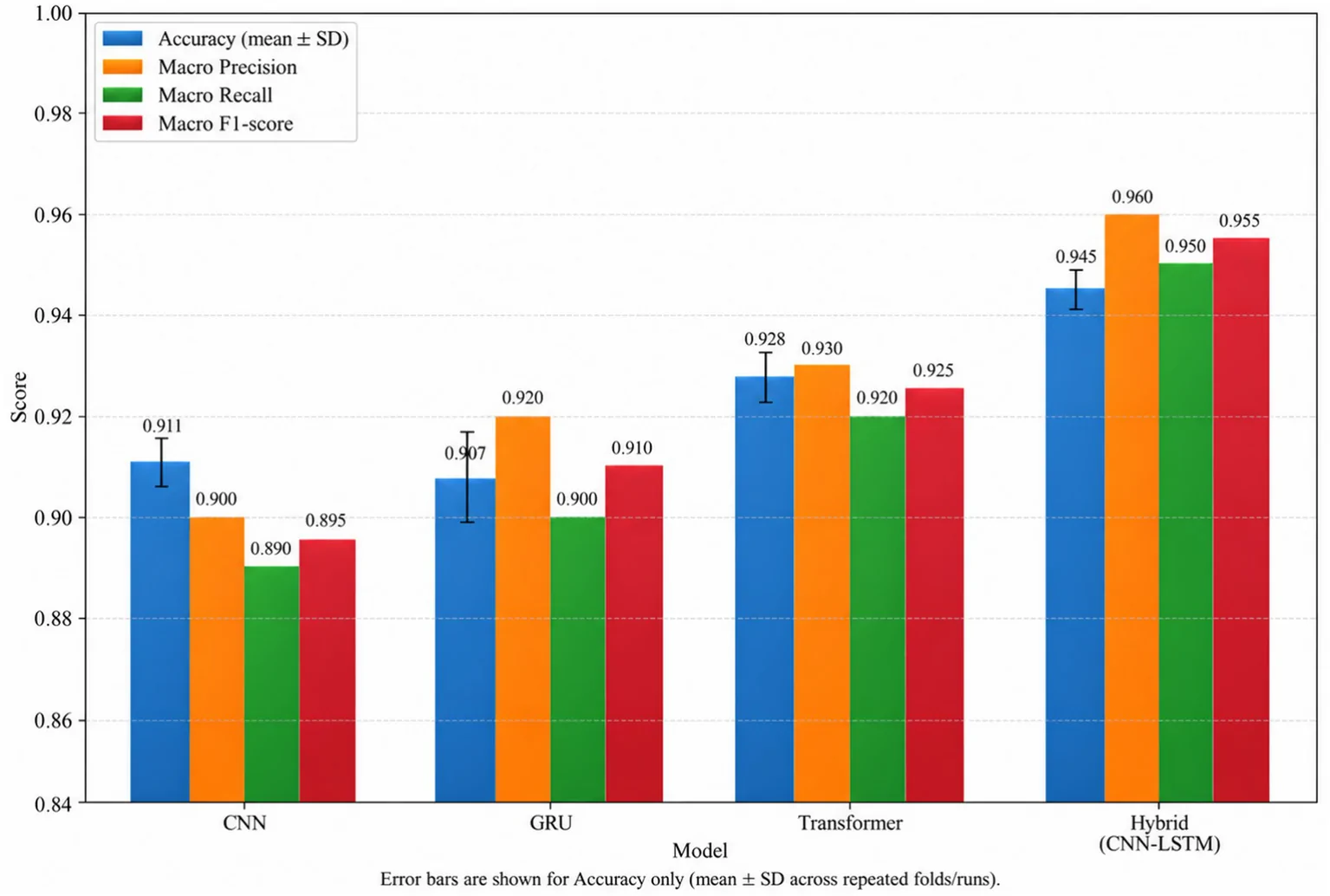
Clean cross-validation performance of representative DL architectures on CICMalDroid 2020.

### 4.2 Reliability and stability

To analyze the detection reliability at the class level, the models were tested separately on benign applications and the four malware categories: adware, banking malware, SMS malware, and riskware. Figure 5a reports the class-wise results, and the malware false-negative rates are reported separately in Figure 5b to avoid visual compression of the security-critical FNR values onto the same axis as F1-scores. The results show that all models performed comparably well on benign and riskware samples, while adware remained the most challenging category for all the evaluated architectures. This pattern confirms that aggregate performance does not provide a full description of category-level detection reliability in AMD.

**Figure 5:**
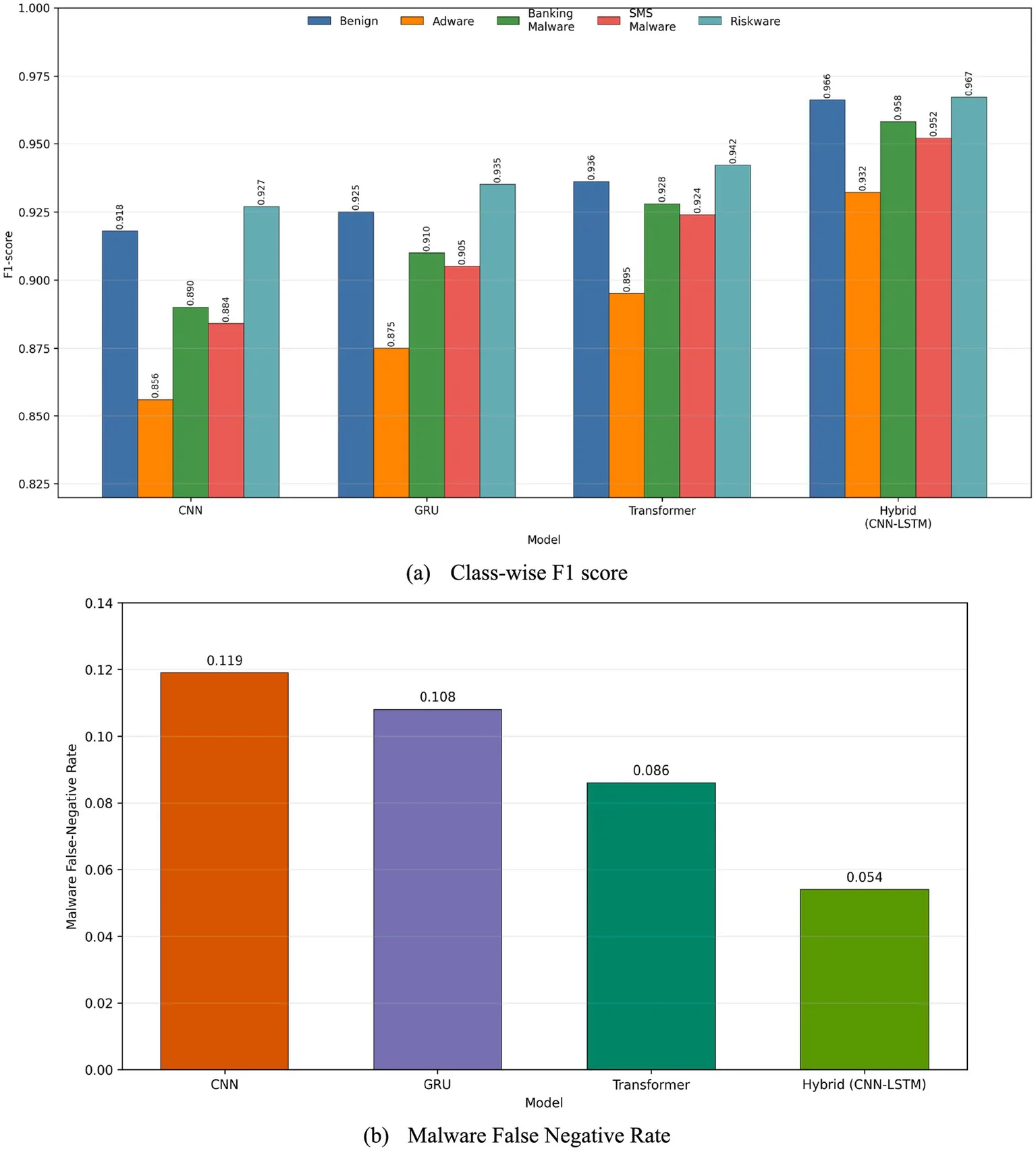
Class-wise malware detection reliability and false-negative behavior of representative DL architectures. **(a)** Reports class-wise F1-scores for benign, adware, banking malware, SMS malware, and riskware categories. **(b)** Reports malware false-negative rate separately to avoid visual compression of the security-critical FNR metric. Lower false-negative rate indicates fewer missed malware samples.

The Hybrid CNN-LSTM model has the best class-wise profile, with F1-scores of 0.966, 0.932, 0.958, 0.952, and 0.967 for benign applications, adware, banking malware, SMS malware, and riskware, respectively. The Transformer model followed with consistently strong class-wise performance, especially for benign, banking malware, SMS malware, and riskware categories. CNN and GRU were competitive but showed weaker performance on adware, confirming that some categories of malware are harder to classify, even when the overall clean benchmark performance looks good. The false negative rate of malware gives a more security-critical look at the reliability of the model, as false negatives are malicious applications that are not detected. As shown in Figure 5b, the lowest malware false-negative rate was obtained using the Hybrid CNN-LSTM (0.054), followed by the Transformer (0.086), GRU (0.108), and CNN (0.119). This shows that the best clean-performance model also has the best malware-miss profile in this experiment. However, these results should be interpreted as class-wise reliability evidence in the specific CICMalDroid 2020 setting, not as evidence of operational reliability on unseen datasets or real-world malware transformations.

Repeated-run stability was evaluated to determine whether the model performance was consistent across independent runs as opposed to a single favorable training outcome. Figure 6 shows the mean macro F1-score with 95% confidence intervals and standard deviation error bars and the corresponding stability level. The Hybrid CNN-LSTM achieved the highest mean macro F1-score of 0.955 with the least variation, followed by the Transformer with 0.925, GRU with 0.910, and CNN with 0.895. Based on this pattern, the Hybrid CNN-LSTM had the strongest and most consistent performance under the shared experimental protocol. But the results are not taken as a definite claim of architecture superiority in all cases, but as evidence from controlled repeated runs. When confidence intervals overlap, or formal pairwise tests are not available, differences in stability should be descriptive rather than conclusive.

**Figure 6:**
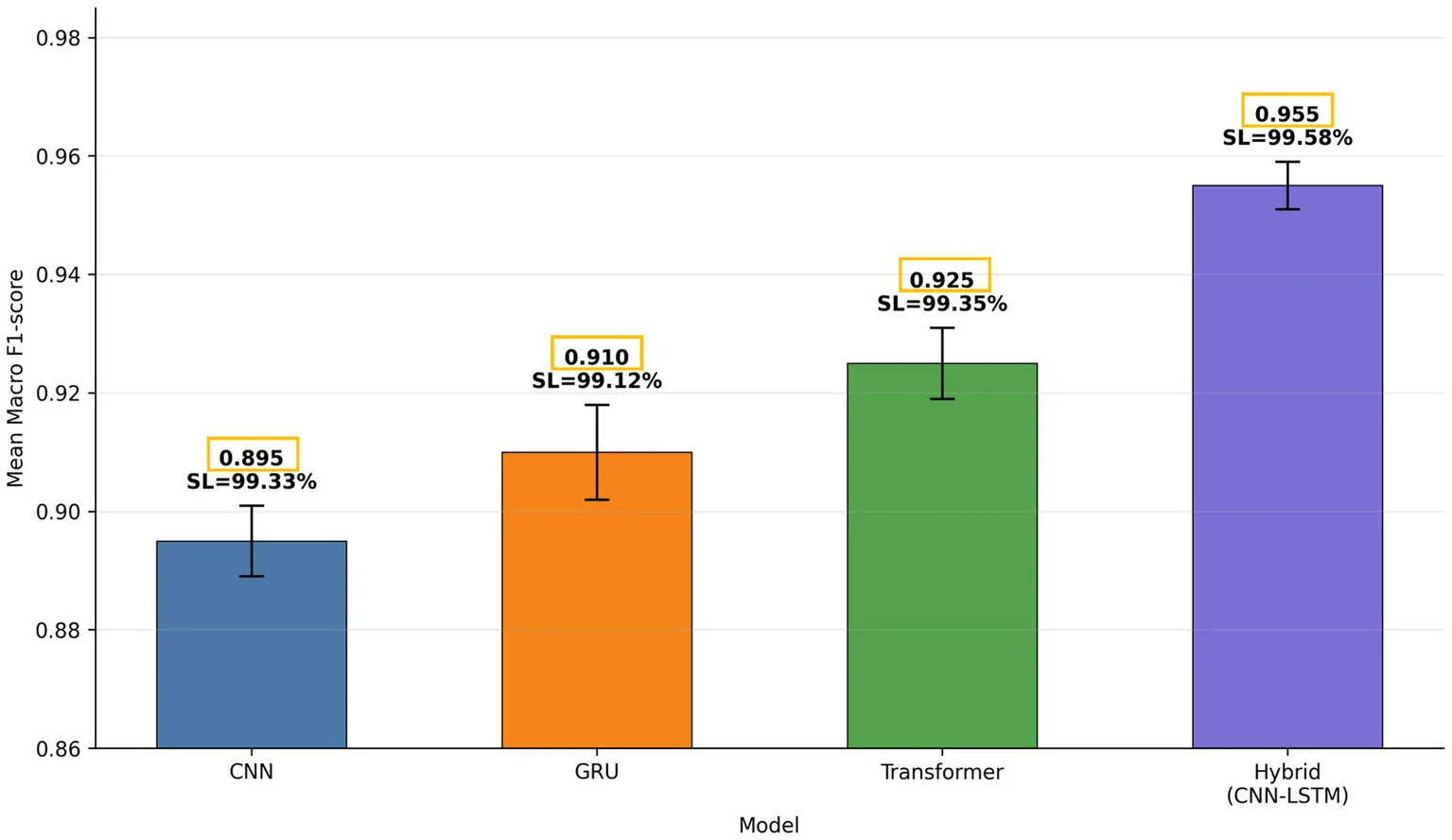
Repeated-run stability of representative DL architectures; the box represents CI and SL = (1 − SD/mean macro F1) × 100.

### 4.3 Feature-masking robustness

Figure 7 illustrates the robustness behavior of the representative DL architectures under multi-level test-time feature masking. To overcome the limitation of using only one masking level, the robustness experiment was extended to 5%, 10%, 20%, and 30% random masking of non-label input features. Models were trained on clean training data, and masking was applied only to the test set. For binary or one-hot encoded features, the selected values were set to 0. For normalized continuous features, the selected values were replaced by the mean of the corresponding feature in the training set. This design makes an evaluation of sensitivity to disturbed feature evidence, not necessarily direct APK-level obfuscation, adversarial code rewriting, repackaging, reflection, or real malware transformation.

**Figure 7:**
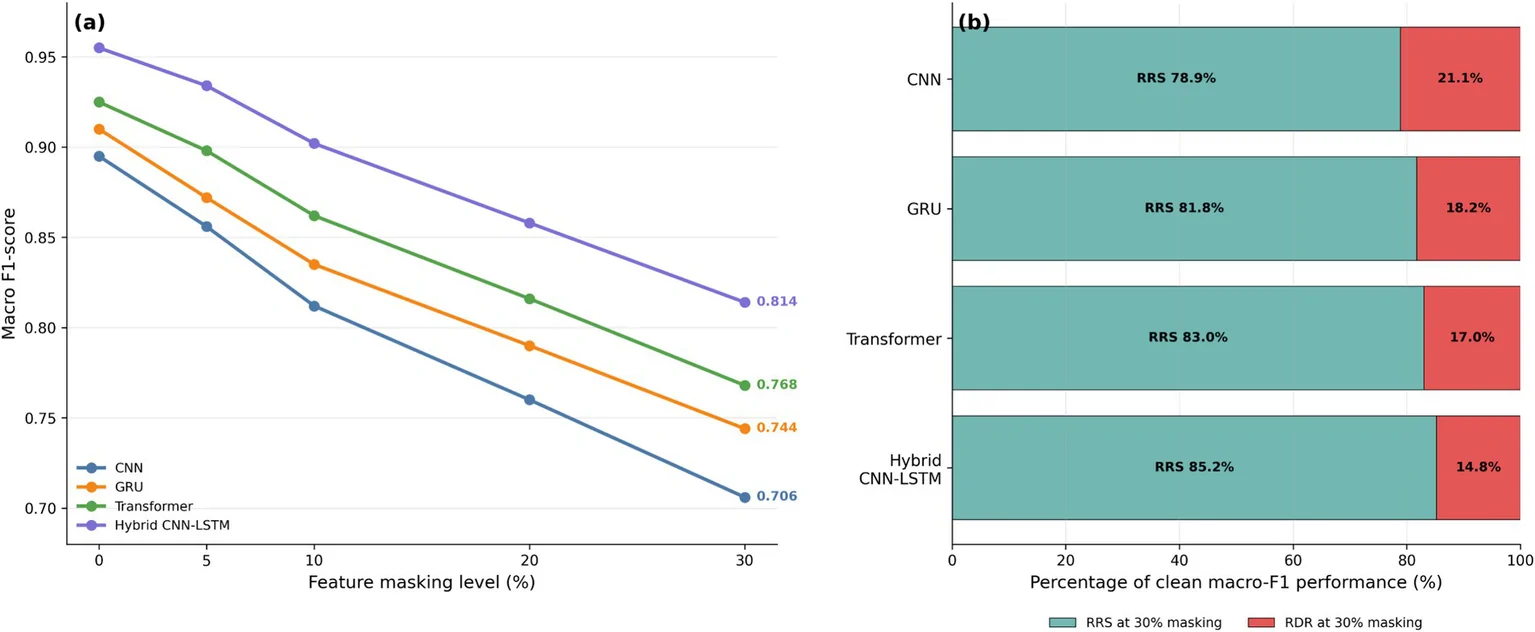
Multi-level **(a)** feature-masking robustness of representative DL architectures on CICMalDroid 2020 and **(b)** Percentage of clean macro F1 performance.

As shown in Figure 7a, the macro-F1 of all models degrades gradually as the masking level increases. CNN suffers the largest drop, going from a clean macro-F1 of 0.895 to 0.706 under 30% masking. GRU drops to 0.744 from 0.910, and Transformer drops to 0.768 from 0.925. The Hybrid CNN-LSTM maintains the best masked-feature performance, falling from 0.955 to 0.814 at 30% masking. This demonstrates that the hybrid spatial-sequential representation is more robust against disturbed feature evidence in the current controlled setting.

Figure 7b summarizes the robustness degradation rate and the robustness retention score at the strongest masking level. CNN has the highest degradation rate of 21.12% and the lowest retention score of 0.789, showing the highest sensitivity to feature masking. The GRU and Transformer show lower degradation rates of 18.24 and 16.97%, respectively. The Hybrid CNN-LSTM has the lowest degradation rate of 14.76% and the highest retention score of 0.852. These results support the main argument that AMD reliability cannot be assessed by clean benchmark performance alone. However, the results should be viewed as controlled evidence for feature-level robustness, not as evidence of resistance to real-world Android malware obfuscation or adaptive adversarial attacks.

Overall, empirical validation (Figures 4–7 data is attached as Appendix A) confirms that clean accuracy is simply a first step for AMD evaluation. All models achieve good results in clean cross-validation, but important architecture-dependent differences emerge in class-wise analysis, malware false-negative evaluation, repeated-run stability, and feature-masking robustness. The results should not be taken as a general ranking of DL architectures for AMD. Instead, they show how the interpretation of models changes when evaluation shifts from aggregate benchmark accuracy to reliability-aware evidence. This directly supports the central contribution of the paper, “DL-based AMD should be evaluated not only in the context of architecture design or clean performance, but also in terms of the credibility and completeness of the evidence supporting reliability claims”.

## 5 Reliability-centered evaluation

The main implication of the study is that recent DL-based AMD is limited not by a lack of new architectures, but by a mismatch between architectural claims and the maturity of supporting evidence. This paper does not propose a new malware detection model. Instead, it proposes a reliability-centered evaluation logic for interpreting DL-based AMD claims. The purpose is to clarify what kind of evidence is required before a model can legitimately claim benchmark performance, class-wise reliability, robustness, explainability, reproducibility, or deployment readiness. Therefore, this section converts the review findings and empirical validation results into a claim-aligned audit framework rather than presenting the evaluated architecture as a new detection system.

### 5.1 Architecture-artifact-validation triad

One major weakness of recent DL-based AMD is the tendency to consider architecture names as directly comparable evidence of progress. This is methodologically brittle as different architectures often work on fundamentally different views of the Android application. A CNN trained on DEX images, a GRU trained on API-call sequences, a Transformer trained on traffic tokens, and a GNN trained on function-call graphs are not simply different models for the same evidence. They represent different evidence systems; each highlights some aspects of malicious behavior while hiding others. This leads to an architecture-evidence mismatch. The reported accuracy may reflect true malware-discriminative learning, but may also reflect artifact encoding choices, dataset age, family overlap, duplicate leakage, preprocessing artifacts, class imbalance, or favorable train-test splits. For image-based models, visual separability may be helpful for fast screening, but it does not automatically prove that the model has learned meaningful malware semantics. Sequence- or traffic-aware models may provide stronger behavioral evidence, but only if the trace captures actual malicious execution instead of sandbox-specific or incomplete behavior. For graph- and attention-based models, richer representation capacity raises rather than lowers the evidential burden: graph construction, attention grounding, and computational feasibility have to be demonstrated rather than assumed.

The main implication is that the right unit of evaluation is not the architecture alone, but the architecture–artifact–validation triad. The architecture determines how evidence is learned, the artifact representation determines what Android evidence is made available, and the validation protocol determines if the learned evidence is credible beyond a favorable benchmark setting. Without that triad, comparing architectures can be superficial. A higher score may indicate a better detector, but it may also indicate easier artifacts, weaker validation, or dataset-specific regularities. This triadic view immediately accounts for the results of Sections 3 and 4. The evidence matrix showed that many studies are relatively complete in reporting datasets, features, architecture, and standard metrics, but less complete in proving robustness, interpretability, reproducibility, and deployment readiness. This view of the triadic explains why the empirical validation presented here should be considered as a reliability demonstration and not as a ranking of universal architectures. The evaluated CNN, GRU, Transformer, and Hybrid CNN-LSTM models were used to demonstrate the changes in the interpretation of the model when the accuracy is complemented with the class-wise reliability, malware false-negative behavior, repeated-run stability, and multi-level feature-masking robustness. They are not presented as a new family of models, nor are the results meant to prove general superiority on all AMD datasets and deployment contexts.

### 5.2 Claim-aligned audit

The architecture-evidence mismatch calls for a stricter evaluation logic. The problem is not simply that future studies should report more metrics; rather, each reliability claim requires a corresponding level of evidence. A model that reports clean accuracy can claim benchmark discrimination, but not robustness or deployment claims. A model that reports class-wise recall can make a stronger case for malware-category reliability, but not necessarily for operational feasibility. Similarly, a model that reports latency and memory cost cannot be considered deployment-ready unless robustness, false negatives, feature stability, interpretability, and update feasibility are also addressed. Therefore, this study introduces a “claim-aligned reliability audit” presented in Table 11 for DL-based AMD. The novelty of the audit is that each layer of evaluation is matched with the type of claim it can legitimately support. This changes model comparison from a simple accuracy-ranking to a reliability-centered assessment. Conventionally, the model with the highest accuracy score is considered best. However, from a reliability-centered perspective, the best-performing model under the shared protocol is the one whose claims are supported by the relevant layers for its intended use. Not every study needs to strictly satisfy every layer, but it should clarify that the strength of a claim should match the strength of supporting evidence. The proposed audit also prevents overclaiming. An XAI heatmap is not necessarily an explanation unless mapped to meaningful Android artifacts. A lightweight detector is not deployable unless operational constraints are measured. Similarly, a hybrid model is not necessarily better unless ablation shows which modality contributes to the improvement. Consequently, a model performing well on one dataset cannot claim robustness to others without temporal, family-aware, cross-corpus, or perturbation-based testing.

**Table 11:** Claim-aligned reliability audit checklist for DL-based android malware detection.

Reliability layer	Main risk exposed	Minimum evidence required	Claim authorized
Clean benchmark evidence	High accuracy may be mistaken for reliability.	Accuracy, precision, recall, macro F1-score, confusion matrix.	Baseline benchmark discrimination.
Dataset validity	Performance may reflect dataset age, label noise, duplicate leakage, family bias, or benchmark artifacts.	Dataset provenance, collection period, sample/family distribution, benign-source verification, duplicate removal, labeling procedure (Jo et al., 2023; Shar et al., 2023; Yao et al., 2025; Yapici, 2025; Kardgar et al., 2026).	Dataset-credible benchmark evidence.
Validation credibility	Random splits may inflate performance through temporal leakage or family overlap.	Temporal split, family-wise separation, cross-dataset testing, external validation (Ullah et al., 2024; Yao et al., 2025; Alfaw et al., 2025; Kardgar et al., 2026).	Generalization-aware evaluation.
Class-wise security reliability	Minority/operationally important malware may be poorly detected despite high aggregate accuracy.	Class-wise F1-score, malware recall, false-negative/positive rate, family-level reporting (Tasyurek and Arslan, 2023; Shar et al., 2023; Shen et al., 2023; Alfaw et al., 2025).	Threat-category reliability.
Robustness/evasion resistance	Models may fail under obfuscation, repackaging, feature perturbation, adversarial manipulation, or sandbox evasion.	Clean, perturbed, obfuscated, adversarial, temporal, or cross-dataset performance reported separately (Ullah et al., 2024; Yadav et al., 2022; Bakır, 2025; Laudanna et al., 2025).	Robustness beyond clean benchmark conditions.
XAI/artifact grounding	Heatmaps, saliency, or attention maps may be mistaken for meaningful explanations.	Explanation mapped to Android artifacts (Ullah et al., 2024; Ullah et al., 2022a; Jo et al., 2023; Cai et al., 2021).	Analyst-interpretable evidence.
Reproducibility support	Results may be difficult to verify or reproduce.	Code, data access, preprocessing details, split files, hyperparameters, random seeds, software environment.	Repeatable empirical evidence.
Deployment readiness	A model may be lightweight in design but not operationally deployable.	Model size, latency, memory footprint, preprocessing cost, hardware setting, energy cost, update feasibility, real-device or edge testing (Tasyurek and Arslan, 2023; Shen et al., 2023; Ullah et al., 2022b).	Operational feasibility.

The implication for deployment is that practical AMD may require multi-stage evaluation and deployment rather than reliance on a single architecture. Scalable first-stage screening may be supported by CNN and lightweight models. Deeper behavioral or structural analysis of uncertain or high-risk samples may be supported by sequential, graph-based, transformer, or hybrid models. However, each stage has to be judged in terms of the evidence it claims to provide, including class-wise reliability, robustness, interpretability, reproducibility, and operational feasibility. It changes the focus from choosing the highest-scoring architecture to finding the architecture that has sufficient support for a given security role. The claim-aligned reliability audit is summarized in Table 11. The table is not intended to suggest that every study or current empirical validation meets all deployment-level requirements, but rather to determine what level of evidence is required for each type of AMD claim.

### 5.3 Self-audit of the present empirical validation

To prevent overclaiming, the empirical part of the study was also subjected to the reliability audit that was proposed. The controlled validation on CICMalDroid 2020 provides valuable evidence for baseline benchmark discrimination, class-wise malware reliability, repeated-run stability, and feature-level robustness under multi-level test-time masking. The revised evaluation reports the accuracy, macro precision, macro recall, macro F1-score, standard deviation, confidence intervals, malware false-negative rates, and robustness degradation/retention scores. Thus, the empirical part reinforces the main argument that clean accuracy alone is not sufficient for the assessment of the reliability of DL-based AMD.

However, the empirical validation should not be taken as a full deployment-ready AMD system or as a generic ranking of CNN, GRU, Transformer, and Hybrid CNN-LSTM architectures. However, the work is limited by the fact that it utilizes a single public dataset, shares a hyperparameter protocol, performs feature-level perturbation rather than APK-level obfuscation, adversarial code rewriting, repackaging, reflection, dynamic payload loading, or real-device deployment. The shared training protocol increases comparability across architectures but does not guarantee that each model reached its individually optimized performance ceiling. Similarly, multi-level feature masking tests sensitivity to disturbed feature evidence but does not fully reproduce adaptive malware evasion.

Thus, the empirical part is a controlled reliability-aware validation with moderate-to-strong internal evidence, but not fully operational evidence, according to the evidence-credibility logic proposed in this study. It supports claims about how model interpretation changes with the addition of reliability layers but does not support broad claims of cross-dataset generalization, real-world adversarial robustness, or production deployment readiness. The self-audit is consistent with the aim of the study, which is to demonstrate that AMD claims must be consistent with the evidence provided.

## 6 Conclusion

This study reviewed the recent architecture-centered and reliability-aware DL-based AMD research. The research does not propose a different malware detection model; instead, it instead investigates how architecture families, Android artifacts, and validation practices impact the credibility of reported AMD performance. The review indicated that CNN/image-based models, sequential architectures, graph-based models, transformer/attention-based methods, hybrid systems, lightweight models, and XAI-oriented approaches have all been employed to advance AMD. However, reporting accuracy alone is not enough to judge real detection reliability, as performance depends on how artifacts are represented, the transparency of the dataset, the validation design, robustness testing, reproducibility, and evidence of deployment. This was supported by the controlled empirical validation on CICMalDroid 2020. Under the shared evaluation protocol, the Hybrid CNN-LSTM achieved the best clean-performance profile with an accuracy of 0.945 ± 0.004 and macro F1-score of 0.955, followed by Transformer, GRU, and CNN models. But the results also suggest that clean benchmark performance is only a partial indicator of model reliability. Class-wise analysis showed that adware was the most challenging category among the evaluated architectures. Malware false-negative analysis showed important differences in missed malware detection. The Hybrid CNN-LSTM resulted in the lowest malware false-negative rate of 0.054, followed by the Transformer, GRU, and CNN.

Repeated-run and robustness analyses further confirmed the need for reliability-centered evaluation. Hybrid CNN-LSTM achieved the highest numerical stability level at 99.58%, followed by Transformer, CNN, and GRU. The multi-level feature-masking analysis also showed a gradual decrease in performance as the masking increased (from 5 to 30%), with the Hybrid CNN-LSTM having the highest robustness retention score at the highest masking level. These results show that testing AMD models on just aggregate clean accuracy can hide important differences in class-level behavior, malware-miss risk, run-to-run variation, and sensitivity to disturbed feature evidence. To summarize, the study suggests a claim-aligned reliability audit in DL-based AMD research. The findings indicate that future AMD studies should match their claims to the evidence they actually provide. While benchmark accuracy is a basic measure of classification performance, higher claims of reliability, robustness, explainability, reproducibility or readiness for deployment require additional evidence, including but not limited to class-wise metrics, false-negative analysis, repeated-run reporting, temporal or cross-dataset validation, adversarial or obfuscation-aware testing, transparent implementation details, and evaluation relevant to deployment. Future work should thus move beyond accuracy-centered reporting and design AMD evaluation protocols that better represent real Android malware evolution, operational constraints, and security-critical failure modes.

### 6.1 Limitations

The section explains this study’s limitations. First, the review focused mainly on studies published between 2021–2026 to capture the recent architecture and evaluation trends. Earlier foundational studies were used only where needed for background, method comparison, or relevant support. Therefore, this review should be interpreted as a recent, architecture-oriented, and evidence-centered review rather than a comprehensive historical survey of AMD. Second, the studies considered differ substantially in terms of datasets, malware families, benign sources, artifact types, feature extraction methods, validation protocols, and reported metrics. Due to this heterogeneity, a direct numerical comparison of the accuracy values is methodologically unreliable. Different trained models could not be tested on the same detection settings.

Third, evaluating the evidence partly depends on the reporting quality of the original studies. Some studies gave full methodological details, while others reported few details. Finally, we conducted the controlled empirical validation on CICMalDroid 2020 using four representative architectures. The results show how interpretation changes under claim-aligned reliability evaluation but should not be considered as a universal ranking of DL architectures. The feature-masking robustness remains a feature-level stress test, not APK-level obfuscation/adversarial transformation.

The empirical validation should also be viewed within its limitations. First, the experiment was carried out on a single public dataset, CICMalDroid 2020; therefore, the results do not reflect cross-dataset, temporal, or family-wise generalization. Second, the evaluated CNN, GRU, Transformer, and Hybrid CNN-LSTM architectures were trained under a common hyperparameter protocol to support controlled comparison, but this does not guarantee individually optimized performance for each architecture. Third, the multi-level feature masking experiment measures feature-level sensitivity under disturbed input evidence but does not replicate APK-level obfuscation, adversarial code rewriting, repackaging, reflection, dynamic payload loading, sandbox evasion, or real-device deployment conditions. Therefore, the empirical part should be seen not as a complete operational AMD system or a general ranking of DL architectures, but as a controlled reliability-aware validation.

### 6.2 Future research directions

The future research on DL-based AMD needs to evolve in three main directions. First, benchmark construction and validation protocols should be more realistic. Datasets should include collection periods, malware family distributions, benign source verification, labeling procedures, information, duplicate removal strategies, and family overlap controls. Validation should move beyond random train-test splits toward temporal, familywise, external validation and cross-corpus testing.

Second, reliability evidence should be routinely included in AMD evaluation. Future studies should report class-wise F1-score, malware recall, false negative rate, confusion matrices, repeated-run stability, and robustness under Android-specific disturbances. All results (clean, perturbed, obfuscated, temporal, and cross-corpus results) should be reported separately rather than collapsed into a single headline score.

Third, explainability, reproducibility, and deployment readiness should be considered core evidence dimensions. XAI should be mapped to inspectable Android artifacts. To improve reproducibility, code, preprocessing scripts, dataset identifiers, split files, hyperparameters, and feature extraction pipelines should be released wherever possible. Studies that focus on deployment should report model size, inference latency, memory footprint, preprocessing cost, hardware platform, energy impact, update feasibility, and real-device or edge feasibility.

In summary, future work on DL-based AMD should move from isolated benchmark optimization toward transparent, robust, explainable, reproducible, and deployment-aware systems. Progress should therefore be measured not only by architectural novelty or benchmark accuracy, but also by the maturity of the evidence.

## Data Availability

Publicly available datasets were analyzed in this study. This data can be found at: https://ieee-dataport.org/documents/android-malware-detection-dataset, https://www.kaggle.com/datasets/hasanccr92/cicmaldroid-2020, https://www.unb.ca/cic/datasets/maldroid-2020.html.
