# Supplementary material for: Deep learning-based android malware detection under realistic evaluation constraints: an architecture-centric review and reliability-aware empirical validation
Source: Front Artif Intell. 2026 Oct 14;9:1964400. doi: 10.3389/frai.2026.1964400 (PMC13713746; doi:10.3389/frai.2026.1964400)
Supplement: Supplementary file 1 [file Data_Sheet_1.pdf]

Appendix A

(Numerical Data Supporting Figures 4–7)

Figure 4: Clean Cross-Validation Performance

Model	Accuracy, mean ± SD	Macro Precision	Macro Recall	Macro F1-score	Macro F1 95% CI
CNN	0.911 ± 0.005	0.900	0.890	0.895	0.888–0.902
GRU	0.907 ± 0.008	0.920	0.900	0.910	0.900–0.920
Transformer	0.928 ± 0.006	0.930	0.920	0.925	0.918–0.932
Hybrid CNN-LSTM	0.945 ± 0.004	0.960	0.950	0.955	0.950–0.960

Figure 5(a): Class-Wise F1-Score

Model	Benign	Adware	Banking Malware	SMS Malware	Riskware	Macro F1
CNN	0.918	0.856	0.890	0.884	0.927	0.895
GRU	0.925	0.875	0.910	0.905	0.935	0.910
Transformer	0.936	0.895	0.928	0.924	0.942	0.925
Hybrid CNN-LSTM	0.966	0.932	0.958	0.952	0.967	0.955

Figure 5(b): Malware Macro-Recall and False-Negative Rate

Model	Malware Macro-Recall	Malware False-Negative Rate
CNN	0.881	0.119
GRU	0.892	0.108
Transformer	0.914	0.086
Hybrid CNN-LSTM	0.946	0.054

Figure 6: Repeated-Run Stability

Model	Mean Macro F1	SD	95% CI	Stability Level (SL, %)
CNN	0.895	0.006	0.888–0.902	99.33
GRU	0.910	0.008	0.900–0.920	99.12
Transformer	0.925	0.006	0.918–0.932	99.35
Hybrid CNN-LSTM	0.955	0.004	0.950–0.960	99.58

Figure 7: Multi-Level Feature-Masking Robustness

Model	Clean F1	5% Masked F1	10% Masked F1	20% Masked F1	30% Masked F1	RDR at 30% (%)	RRS at 30% (ratio)
CNN	0.895	0.856	0.812	0.760	0.706	21.12	0.789
GRU	0.910	0.872	0.835	0.790	0.744	18.24	0.818
Transformer	0.925	0.898	0.862	0.816	0.768	16.97	0.830
Hybrid CNN-LSTM	0.955	0.934	0.902	0.858	0.814	14.76	0.852

Notes: SD = standard deviation; CI = confidence interval; SL = Stability Level; RDR = robustness degradation rate; RRS = robustness retention score. SL was computed as SL = (1 − SD / mean macro F1) × 100. RDR was computed as ((clean F1 − masked F1 at 30%) / clean F1) × 100. RRS was computed as masked F1 at 30% / clean F1.
